# Real-Time Localization of Moving Dipole Sources for Tracking Multiple Free-Swimming Weakly Electric Fish

**DOI:** 10.1371/journal.pone.0066596

**Published:** 2013-06-21

**Authors:** James Jaeyoon Jun, André Longtin, Leonard Maler

**Affiliations:** 1 Department of Physics, University of Ottawa, Ottawa, Canada; 2 Department of Cellular and Molecular Medicine, University of Ottawa, Ottawa, Canada; 3 Center for Neural Dynamics, University of Ottawa, Ottawa, Canada; Cornell University, United States of America

## Abstract

In order to survive, animals must quickly and accurately locate prey, predators, and conspecifics using the signals they generate. The signal source location can be estimated using multiple detectors and the inverse relationship between the received signal intensity (RSI) and the distance, but difficulty of the source localization increases if there is an additional dependence on the orientation of a signal source. In such cases, the signal source could be approximated as an ideal dipole for simplification. Based on a theoretical model, the RSI can be directly predicted from a known dipole location; but estimating a dipole location from RSIs has no direct analytical solution. Here, we propose an efficient solution to the dipole localization problem by using a lookup table (LUT) to store RSIs predicted by our theoretically derived dipole model at many possible dipole positions and orientations. For a given set of RSIs measured at multiple detectors, our algorithm found a dipole location having the closest matching normalized RSIs from the LUT, and further refined the location at higher resolution. Studying the natural behavior of weakly electric fish (WEF) requires efficiently computing their location and the temporal pattern of their electric signals over extended periods. Our dipole localization method was successfully applied to track single or multiple freely swimming WEF in shallow water in real-time, as each fish could be closely approximated by an ideal current dipole in two dimensions. Our optimized search algorithm found the animal’s positions, orientations, and tail-bending angles quickly and accurately under various conditions, without the need for calibrating individual-specific parameters. Our dipole localization method is directly applicable to studying the role of active sensing during spatial navigation, or social interactions between multiple WEF. Furthermore, our method could be extended to other application areas involving dipole source localization.

## Introduction

Animals must accurately locate signal sources of various kinds [1]–[5], since this allows animals to quickly respond to prey, predators, or potential mate signals that are very important for their survival. Mechanisms of signal localization depend on particular signal types, but in general, signals attenuate over distance; therefore the intensity information can be useful in signal localization. In fact, many biological and non-biological systems can determine signal locations on the basis of comparing received signal intensities (RSI) from two or more spatially distributed sensors [2], [6]–[8]. The signal localization task becomes more complex if the RSI depends additionally on the orientation of the signal source, such as the signals dependent on the orientation of an electric fish dipole. A current dipole consists of two physically separated current sources having opposite polarities, which creates spatially non-uniform field strength as a function of distance and orientation. A dipole source can be approximated as an ideal dipole if the separation between the positive and negative sources is much smaller than the distance between the source and the detector [9]. Animals transmit or receive dipole-like signals in the form of sound [2], [3], vibration [4], [10], and electricity [1], [11]. For example, weakly electric fish species can locate other member of species using the electric field they generate [12]–[16].

It is necessary to track free-moving weakly electric fish (WEF) to study their electrolocation or social behaviors under naturalistic conditions. Visual tracking is widely employed for studying animal behaviors, and a typical setup requires an appropriate illumination and a background to produce high contrast images. Animal tracking can be automated by a computer vision algorithm [17]–[24], but visual tracking algorithms become less reliable in visually complex scenes [7], [25]. Animals’ naturalistic habitats often contain objects or other animals, which can cast shadows, obstruct, or confuse the identity of the animal being tracked. Use of visual markers [17], [18], [19] or electronic tags [7], [25]–[27] can improve the tracking reliability during animals’ interaction with objects or with other animals. In particular, electrical tracking methods do not require a direct line of sight, thus the tracking remains reliable while animals are obstructed from view. Here we propose a method of locating current dipole sources in order to track multiple freely swimming electric fish in an environment with objects and/or other electric fish.

WEF are mostly nocturnal and often found in turbid water, thus their visual sensing range is limited in their natural habitat. Instead, they generate an electric field using one or more electric organs in order to perceive their immediate sensory surroundings [6], [13], [28]–[31], or to communicate between individuals [1], [12], [32]–[37]. The geometry of the fish electric field is dipole-like since the rostral and caudal parts of the electric organs generate current flows in opposite directions [38]–[40], and they closely approximate an ideal dipole model at a distance scale greater than fish’s body length [9]. The waveform of the electric organ discharge (EOD) is either continuous or pulsatile depending on the species, and the extent of the electric organ is either concentrated or dispersed along the rostro-caudal axis [41]. In the Gymnotid species we study [28], [42]–[44], the EOD pulses are 1–2 milliseconds in duration, and multiple components of the electric organ activate at different pulse phases to create a complex spatiotemporal pattern [39], [40]. When two or more fish are nearby, they discharge so as to actively avoid the collision of their pulses, thus minimizing signal interference between individuals [45]–[47]. Although the localization of dipole sources is complicated by the presence of multiple current sources distributed along the body, and occasional signal interference between animals, it is possible to solve the dipole localization problem by optimizing the EOD pulse measurement and by using the ideal dipole model.

In this paper, we propose a dipole localization method based on the lookup table (LUT) operation, which compares actual received signal intensities from multiple recording channels with theoretically predicted signal intensities at many possible dipole locations. Our LUT search algorithm was computationally optimized for real-time tracking of a freely swimming WEF in a shallow tank to determine its position, orientation, and body-bending angle. In addition, our tracking system reliably dissociated the trajectories of fish dyads and their EOD pulses. Our dipole tracking system could be useful for field studies where long-term visual observation is difficult [48], [49]; or it could be used in conjunction with visual tracking to study social interactions in a visually complex environment. Our unique approach to the inverse problem using an ideal dipole model could also be applicable to other dipole localization problems of such as sound or vibration source localizations.

## Methods

### Overview

**Figure 1:**
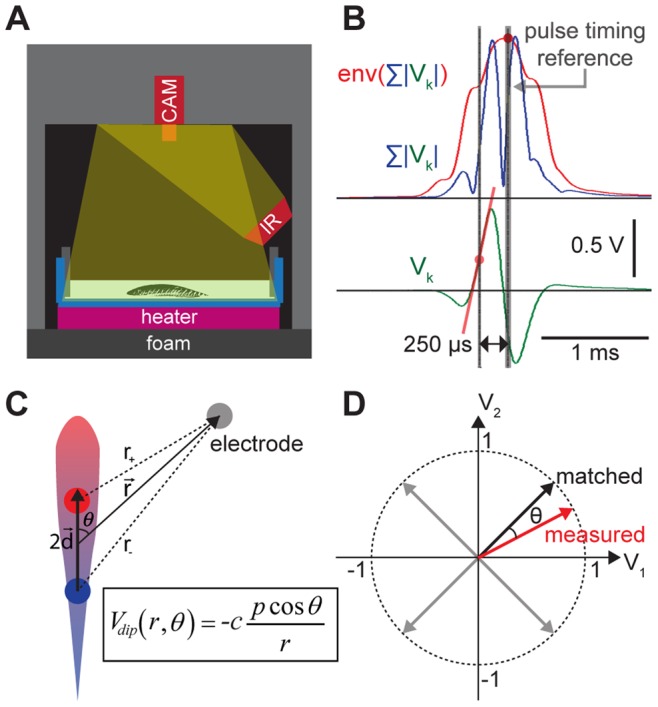
EOD signal measurement and ideal dipole approximation. **A**) Our experimental setup. Electrodes were attached on the tank wall, and concurrent video recordings were made under infrared illumination. **B**) Received signal intensity (RSI) measurement. The original waveforms (green) were rectified then summed from all channels (blue). Signal envelope (red curve) was extracted using an RMS filter, and a pulse timing reference (thick grey) was determined at the peak. An instantaneous slope of the original waveform was measured at 250 µsec before the reference timing (red line). **C**) Ideal dipole voltage (*V_dip_*) approximation of an electric fish in two dimensions. **D**) Lookup table search using a dot-product to find the best matching vector.

We measured the EOD pulse amplitudes at multiple locations with respect to multiple fixed detectors in shallow water (Fig. 1A), and fitted them with an ideal dipole model by varying the position and orientation parameters. The instantaneous slopes at a particular EOD pulse phase were measured from all channels of the detector pairs when the current flow was concentrated near the center of the fish’s body [39]. The RSIs were more accurately measured with the instantaneous slopes than the peak-to-peak amplitudes, because the slope measurements were taken nearly instantaneously (120 µsec) and concurrently from all channels in a phase-locked manner. Due to the brief measurement duration, the slope measurement was less susceptible to the signal interferences between WEFs, and it agreed with the ideal dipole model more closely due to a closer physical separation between the current sources when the measurements were taken. The slope measurements were made relative to the pulse timing reference, which was defined at the peak of the global pulse envelope (Fig. 1B). The pulse envelope measures the total power received by all recording channels, and was calculated by first summing all channels after rectification (blue trace in Fig. 1B), then smoothing to have a unimodal shape with only a single peak (red trace in Fig. 1B) [50]. The measured RSI values were compared with the theoretical values computed by the two-dimensional ideal dipole model (Fig. 1C) at many possible dipole locations, and the closest matching location was found by our search algorithm (Fig. 1D). Although it is possible to observe similar normalized RSIs from two different dipole locations, our use of eight recording channels made this highly unlikely. Our dipole search algorithm initially estimated a location in coarse grids using pre-computed values, and it subsequently searched in finer grids around the initial estimate. This two-step search procedure quickly found a dipole location without compromising spatial or angular resolutions. We achieved a further speed increase (∼1000 localizations/sec) after applying a series of optimization techniques.

### Experimental Setup

All experiments described in this paper were approved by the University of Ottawa Animal Care Committee (protocol number: CMM-143). The animal experiments were conducted in a shallow, circular tank (1.5 m diameter, 10±2 cm water depth), which was surrounded by an enclosure to block external sources of light and electrical noise. We obtained South American pulse-type weakly electric fish *Gymnotus* sp. [28], [42], [51] (species and gender unknown) from a local supplier, and conditioned the aquarium water similarly to their natural habitat (100±20 µS/cm, pH 7±1, and 25±1°C) using a stock salt solution [2] and a floor heater (ThermoTile; ThermoSoft, Buffalo Grove, IL). Animals were active in darkness, thus visual observations were made under near-infrared illumination (850 nm, S8100-60-B/C-IR; Scene Electronics) by a near-infrared sensitive camera (15 frames/sec, 640×480, C910 Logitech, infrared filter removed). The electric organ discharge (EOD) signals were recorded from eight or sixteen vertical graphite electrodes attached on the aquarium wall, and the graphite rods (6 in. long, Mars carbon 2-mm type HB; Staedtler) were coupled to BNC cables (RG54) via heat shrinks. The BNC shielding wires and a signal ground were connected to the Faraday cage which enclosed the whole aquarium (Fig. 1A). Raw signals from the four or eight electrodes pairs were differentially amplified and filtered (200×, 200 Hz ∼ 2.5 KHz, Intronix 2015F; Bolton, Ontario, Canada) to cancel the common-mode noise, and digitized (40 KS/s per channel at 16 bit, CED 1401 mkII; Cambridge Electronic Design, Cambridge, UK). The video recordings were synchronized to the EOD recordings using infrared light pulses (1 msec duration and 10 sec interval), and captured by Spike2 video recorder software (Cambridge Electronic Design, Cambridge, UK).

### EOD Pulse Measurements

We measured the received signal intensities (RSIs) from the recording channels using the slope of the EOD waveform at particular pulse phases, since the slope is proportional to the RSI. We initially used the peak-to-peak amplitudes to measure the RSI, but the measurements deviated from the ideal dipole model due to distortion of the pulse waveform during active body movements, and the ambiguity of determining the waveform polarity. In order to accurately determine the RSIs from all recording channels, the slope measurements from all channels took place synchronously at a fixed time delay from the pulse timing reference, which was defined at the peak of the envelope waveform (Fig. 1B). The envelope waveform (red trace in Fig. 1B) was extracted using a root-mean-square (RMS) filter, (τ = 250 µsec) from the summation of all channels after the rectification (blue trace in Fig. 1B) to prevent signal cancellation. The envelope waveform provided reliable and precise pulse timing reference [50], [52]. In the species we studied, the EOD pulses are initiated near the rostral region and subsequently propagated to the caudal region [39], [53]. By using this knowledge, we found the optimal measurement timing when the electric organ (EO) activation was concentrated at the center of the body (225 µsec before the reference timing), which is the location tracked by a typical visual tracking algorithm. We also found another useful measurement timing when the EO activation was concentrated near the tail location (225 µsec after the reference timing), as this enabled us to deduce the tail-bending angles by comparing the measurements at the central and the tail regions. The instantaneous slopes were measured within a brief time window using five ADC samples (125 µsec duration at 40 KS/s) in order to minimize the probability of the EOD pulses overlapping between different individuals. The slope measurements were performed in Spike2 (Cambridge Electronic Design, Cambridge, UK), and the results were exported to Matlab. A Spike2 script, a sample dataset, and an instruction manual are available in the (Data S1).

### Two-dimensional Ideal Dipole Model

Our experimental data were modeled by considering that the WEF is an ideal dipole in a two-dimensional (2D) space, because 1) fish swam in a shallow body of water, and 2) the measurements were taken by vertically oriented electrodes across the full depth of the water column, which amounts to averaging measurements across depth. The dipole in our model consists of a pair of positive and negative current sources separated by the distance *d* (Fig. 1C). According to Gauss’ law, the potential at a field location 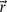 due to such a horizontal dipole 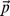 is (see Text S1):
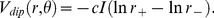
(1)where 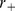 is the distance between the positive source to the field location, and 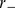 is the distance between the negative source to the field location. *c* is the constant of proportionality which we determined from the three-dimensional boundary condition (see Text S1). In the limit of 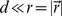, Eq (1) can be approximated as:
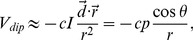
(2)where 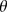 is the angle between the vectors 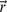 and 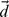. See Text S1 for the detailed derivation of the two-dimensional ideal dipole formula. Although we assumed a simple two-dimensional space without boundaries, Eq (2) closely approximates a more realistic three-dimensional ideal dipole potential computed for a shallow, circular body of water with boundary effects (see Text S1). The top interface between the air and the water, and the bottom interface between the water and the glass contain surface charges induced by the ideal current dipole. The method of image charges can simplify our analysis of the two parallel dielectric interfaces by replacing the surface charges with an infinite number of image current sources (Fig. S1A) [54], [55]. Although the two parallel surfaces generate infinite number of reflections [56], the net potential converges since each successive reflection produces a weaker image current source at a further location (Fig. S1B) [57]. Furthermore, the method of image charges can be extended to determine the potential due to an image dipole. See Text S1 for the detailed description of the three-dimensional ideal dipole potential computed for a shallow, circular body of water.

#### Near field effect correction

The 2D ideal dipole formula did not accurately predict the experimental data when fish were too close to the electrode, where the ideal dipole assumption (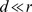) does not hold. However, animals spent a lot of their time near the wall, and this created non-ideality at the near-field location. The electric field nearest to the fish was distorted by its skin conductance, and a channel became saturated when fish made a direct contact to one of the electrodes. In order to ensure accurate predictions by the 2D ideal dipole model, we excluded a channel from being used for our dipole localization procedure when the fish approached one of its electrode pair closer than a set threshold (13 cm).

#### Side boundary effect

A current dipole also induces a surface charge at the interface between the water and the tank wall. The method of image charges can be similarly applied to simplify our analysis by replacing the induced surface charge with a pair of image sources, or an image dipole (Fig. S2C). The circular side boundary forms an image dipole outside of the circular region, and we determined the effect of the side boundary on the voltage difference between a pair of electrodes (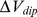). 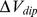
is the quantity we measured to estimate the RSI, and it is the sum of contributions from the dipole (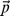) and the image dipole (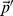): 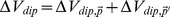. It can be shown that the potential due to the image dipole (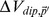) is proportional to the potential due to the dipole (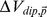) for any dipole location within the circular region (see Text S1) [55]:
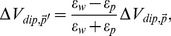
(3)where 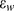 is the permittivity of water, 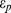 is the permittivity of a plastic wall. Thus, the differential voltage can be simplified to:



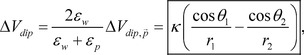
(4)where 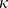 is a constant factor, 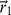 is the vector from the dipole location to the positive electrode, and 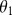 is the angle between the vectors 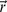
and 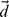. 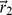 and 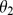 are similarly defined for the negative electrode. In summary, the presence of the circular boundary simply rescales the differential voltage between a pair of electrodes. This scaling effect of the circular boundary does not affect the performance of our dipole localization algorithm, since it uses relative signal intensities between the recording channels.

### Verification of the 2D Ideal Dipole Model with WEF Measurements

In order to experimentally verify our 2D ideal dipole model predictions, we compared the values predicted by the model with differential voltage measurements from a restrained WEF. One fish (23 cm long) was held in a floating platform [50] and positioned 5 cm below water, and the location and orientation of the platform was controlled using a guiding wire installed across the circular wall. We took measurements from four pairs of electrodes with 180° pairing angle at each 5 cm step of translating the animal along the guiding wire from −55 cm to 55 cm, and 15° step of rotating the animal with respect to the guiding wire from 0° to 180°. The position and the orientation of the fish were used to predict the RSI values using the 2D ideal dipole model, and the predicted RSI values were compared against the measured RSI values.

**Figure 2:**
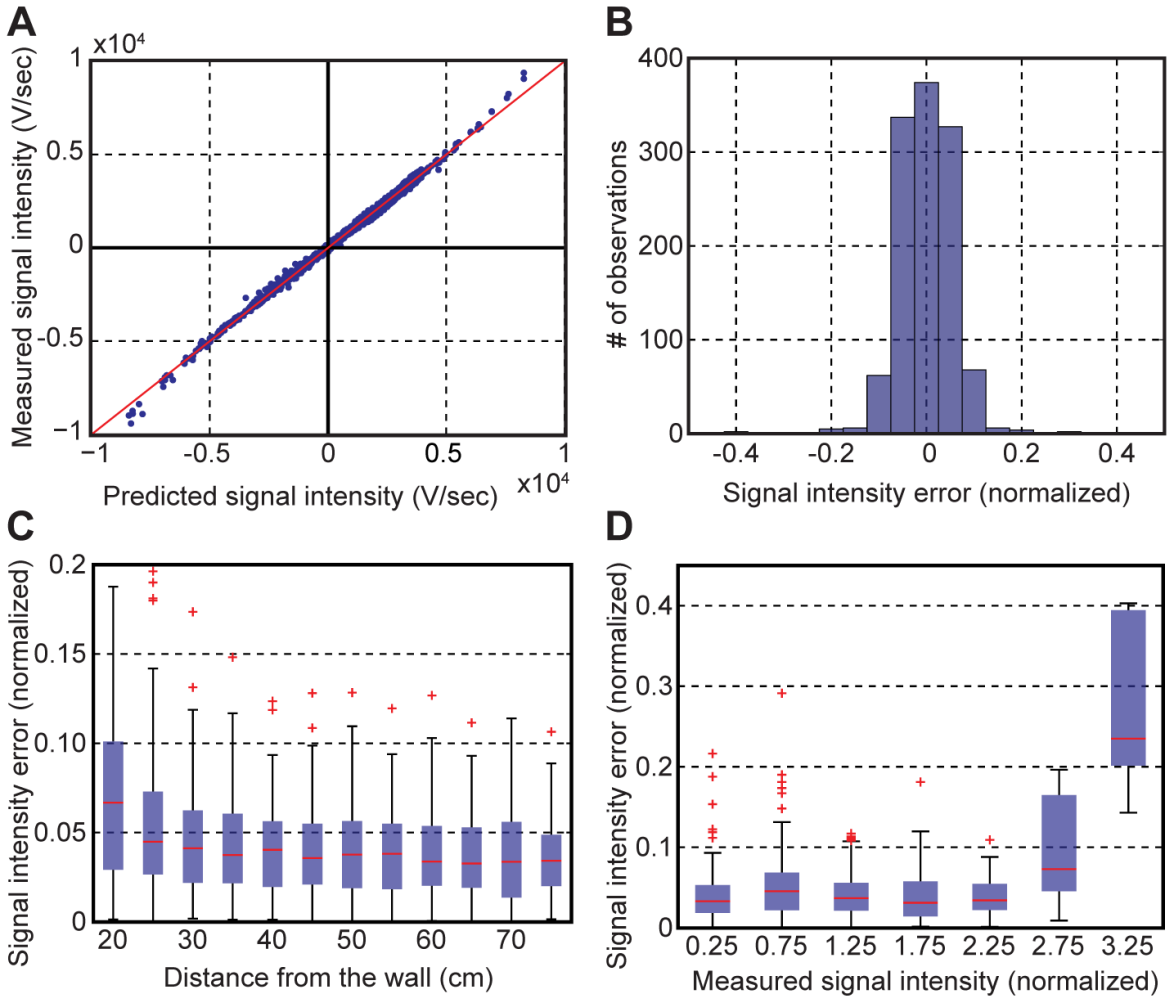
Experimental data fitted by the ideal dipole model. **A**) The measured RSI values (slopes of differential voltages) closely agree (*ρ_corr_* = 0.9983) with the RSI predicted by the ideal dipole model. **B**) The error distribution of the RSI values normalized to the SD of the measurement averaged across the whole tank. The errors were computed by the measured values minus the predicted values. **C**) The RSI error plotted as a function of the distance from the tank wall. The distance was measured to the center of the body. The edges of the boxes represent 25^th^ and 75^th^ percentiles, and the center mark (red line) is the median. Outliers are individually plotted in red. **D**) The normalized RSI error as a function of the normalized measured RSI. The RSI values were each grouped within the range of ±0.25.

Our 2D ideal dipole model accurately predicted the measured RSI from the position and orientation of the restrained animal. There was a tight correlation (*ρ_corr_* = 0.9983, *n* = 1196) and a closely linear relationship (*R^2^* = 0.9966) between the predicted vs. measured RSI (Fig. 2A). The scaling factor was determined by estimating a linear fit to the data. The RSI error was defined as a difference between the observed and the predicted RSI values, and normalized to the SD of the measurement (2706 V/sec). The normalized absolute errors were 0.045±0.038 (mean ± SD), and most of the errors fell within a narrow range (95% of errors less than 0.1) (Fig. 2B). The RSI errors increased when an animal was near the wall (Fig. 2C). The center of the body was always used to measure the distance to the boundary. The greater errors near the boundary were expected from the near field effect, and from the distortion caused by the fish’s skin conductivity. As shown in Figure 2D, the maximum RSI range (3.25±0.25) had the greatest error, which typically occurred when the fish’s head or tail were closest to one of the recording electrodes. In summary, the RSI predicted by the 2D ideal dipole model closely agreed with the actual physiological measurements from WEF at most locations.

### Determining Optimal Electrode Configuration by Simulation

**Figure 3:**
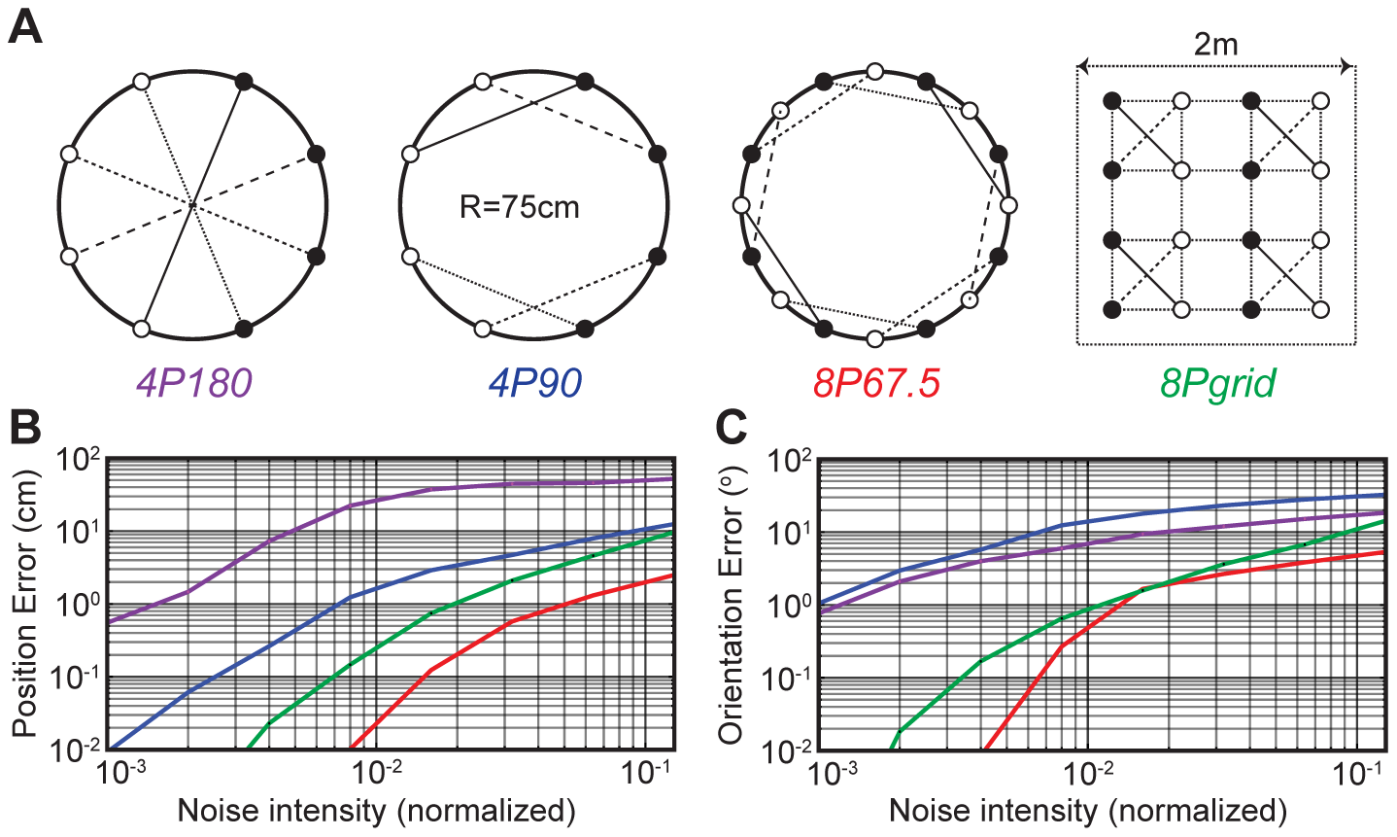
Comparison of the simulated noise performances of the four electrode configurations. **A**) The four types of electrode configurations tested in simulations. Filled circles represent positive electrodes and open circles represent negative electrodes for the differential voltage measurements. Electrodes pairs are connected with lines. **B**) The position errors plotted as a function of the simulated noise intensity (plotted on a log-log scale). The noise intensities were normalized to the SD of the measurement. **C**) The orientation errors plotted as a function of the simulated noise intensity (plotted on a log-log scales).

We simulated four types of electrode configurations and compared their dipole localization performances in the presence of simulated measurement noise. The four electrode configurations we tested varied in the number of channels, the pairing angle, and the geometric layout of the electrodes (see Fig. 3A). The three configurations had a circular geometry (1.5 m in diameter) similar to our experimental tank, and one configuration had a square grid layout (2 m in length, 0.5 m grid spacing) similar to the setup used by Henninger et al. [49]. In our simulation, a dipole location and orientation were randomly assigned at 10 million points, and the RSIs were computed at the recording dipoles according to the 2D ideal dipole model for each electrode configuration. We then simulated the measurement noise by adding random Gaussian noise to the computed RSIs, and provided these values to the dipole localization algorithm. In order to test the effect of noise on the localization accuracy, we varied the noise intensity on a logarithmic scale and normalized the noise intensity to the median of all RSI values across the randomly chosen sampling points. The localization accuracy was quantified by comparing the algorithm-inferred dipole locations with the actual assigned locations.

The localization accuracy was significantly improved when the number of channels doubled from four (*4P90*) to eight (*8P67.5*) as expected from the higher signal-to-noise ratio (SNR) (Fig. 3B). While using only four channels, changing the pairing angle alone from 180° to 90° significantly improved the accuracy (Fig. 3B). Our algorithm also performed accurately for the square grid configuration (*8Pgrid*), which is suitable for field deployment due to the extendable grid layout [49]. Based on our simulations, we implemented the eight-channel configuration (*8P67.5*) for our circular tank using 16 equally spaced perpendicular electrodes. In comparison, the average noise intensity of our actual measurement system was 0.47% of the SD of measured RSI values (averaged across the tank using all locations visited by fish), and the SNR was 46.5 dB.

### Dipole Localization Algorithm Based on LUT Search

We developed an algorithm to deduce the position and orientation of a dipole from a given set of multi-electrode measurements using the 2D ideal dipole model. The measured values were compared with a list of predicted values at many possible locations and orientations of a dipole. The predicted voltages computed by the 2D ideal dipole model were stored in a look-up table (LUT), along with the dipole locations used to compute the predicted voltages. The LUT was constructed once and reused multiple times to increase the search speed. In order to cancel out the constant factor difference between the measured and the predicted RSI values, the RSI measurements from each channel were normalized by the sum of all channels. Thus the LUT stored normalized vectors containing the predicted RSIs at multiple channels. A dipole location was estimated from a given set of measurements by 1) searching for a predicted vector in the LUT having the smallest angle with the given measured vector (Fig. 1D), and 2) the dipole location corresponding to the closest matching vector was retrieved. The angle between the predicted and the measured vectors was determined from their dot product; hence the minimum angle corresponded to the maximum dot product between two unit vectors:
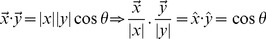
(5)where 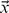 is the given measured vector, and 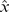 is the normalized vector of 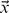. 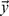 and 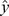 are similarly defined for the predicted vectors in the LUT. 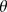 is the angle between 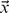 and 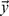. The dot products between the given measured vector and all predicted vectors in the LUT were efficiently computed using a matrix multiplication:
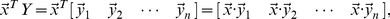
(6)where 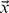 is the given measured vector, 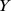 is the matrix containing the predicted vectors (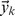) in the LUT, and *n* is the number of entries in the LUT.

We tested various LUT search methods (Table 1) on the data measured from freely swimming WEF (*n* = 11,593). The dot-product based search was compared against the other widely used distance metrics between two vectors (Euclidean distance, city-block distance, etc.) in terms of the speed and accuracy. The dot product metric yielded the highest speed, while the tracking errors were similar between the different search methods tested.

**Table 1:** Comparison of the LUT search methods.

Search methods	speed	Position error (cm)			Orientation error (°)		
	(pulses/s)	*mean*	*median*	*Q90*	*mean*	*median*	*Q90*
Dot product	1068	3.3	1.4	5.0	4.5	4.6	11.0
Cityblock dist.	208	3.3	1.4	5.1	4.4	4.5	10.5
Euclidean dist.	153	3.3	1.4	5.0	4.5	4.6	11.0
Chevychev dist.	77.7	3.4	1.4	5.1	4.6	4.6	10.8
Correlation	44.5	3.5	1.5	5.2	5.2	4.9	12.0

We compared the performances of the five pairwise vector distance metrics used for the LUT search (*90%*: 90^th^ percentile).

### Optimization of the Dipole Search Algorithm

**Figure 4:**
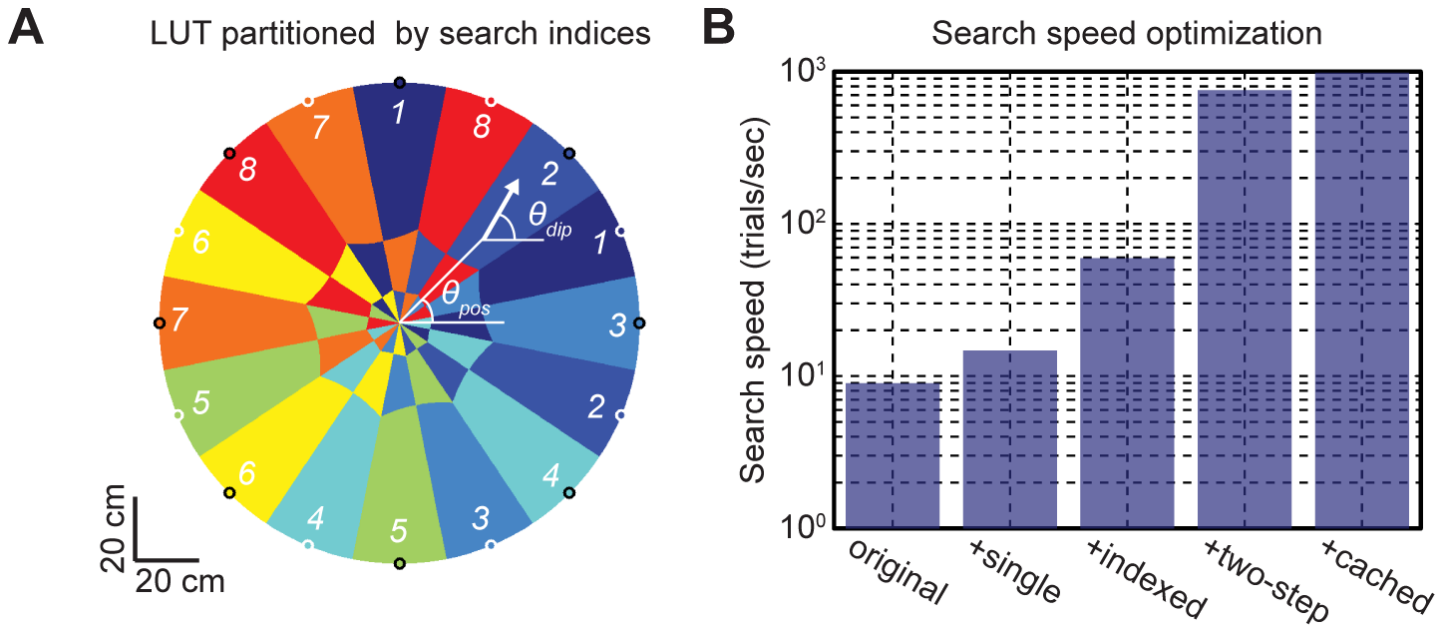
Optimization of the dipole search algorithm. **A**) The circular tank is partitioned by the LUT indices, which are determined by the absolute maximum channels at each dipole location. In this illustration, the orientation of the dipole ( = *θ_dip_*) was set equal to its angular position ( = *θ_pos_*). The electrodes are shown as black (positive) or white (negative) circles, and their fill colors correspond to their channel indices. The channel numbers *1* to *8* correspond to the colors from blue to red as indicated. **B**) Cumulative improvements in the search speed after successively applying the optimization techniques (*original*: single search step, *single*: single numerical precision, *indexed*: LUT indexed by the strongest channel, *two-step*: two-step search procedure, *cached*: fine-grid search was cached with *n_history_* = 16).

In order to implement a practical dipole tracking system, we improved our initial dipole search algorithm up to ∼1000 localizations/sec (running on Intel i7-2720QM 2.2 GHz CPU) by applying a series of computational optimizations. The spatial resolution is determined by the size of a LUT, and increasing the spatial and angular resolutions required larger memory and longer search time. In order to increase the search speed without compromising the spatial resolution, we implemented a two-step search procedure. The first step quickly approximated a dipole location in coarse grids using the LUT, and then the second step found a more accurate dipole location by conducting a search around the initial estimate in finer grids. The coarse grid spacing was set to 2 cm and 4°, and the fine grid spacing was set to 0.5 cm and 1°. We doubled the density of the coarse grid spacing near the wall (within 10 cm) in order to improve the localization accuracy near the boundary. The computational results from previous fine grid searches were stored up to 16 previous histories, and the stored results were reused when the coarse grid search returned the same coordinates as previously encountered. Both the positive and negative dot product ranges were used in order to simultaneously search the parallel and anti-parallel dipole orientations. Our dipole algorithm searched only the locations within the circular boundary, and excluded the cases when the head or tail ends lay outside of the boundary. All numbers were computed in the single-precision format instead of the double-precision format to save the computational time and memory requirements, but without compromising the localization accuracy. The LUT entries were partitioned by their strongest channel indices (Fig. 4A), such that only a matching region (one eighth of the LUT size) had to be searched by using the channel number having the absolute maximum RSI as a filtering criterion. However in the actual implementation, the search also included a region in the LUT where a given channel index was the second strongest, in order to account for possible order reversals induced by noise.

Figure 4B shows cumulative improvements in the search speed after applying each optimization step. The two-step search procedure yielded the highest speed gain, which achieved high localization accuracy without sacrificing speed and memory usage. Our final, optimized search algorithm produced estimates at a rate 20 times the actual EOD pulse rate (∼50 Hz), thus became fast enough to track multiple electric fish in real-time. The LUT cache was built in 0.40±0.01 sec and occupied 30.2 MB of memory using the grid parameters settings described previously. In summary, our optimized dipole tracking algorithm met our practical needs after improving the search speed by a factor of hundred. For the demonstration purpose, an example Matlab code, a sample dataset, and an instruction manual are available in the (Data S1).

### Single Fish and Fish Dyads Tracking

Our dipole localization algorithm was then applied to every EOD pulse measurement from a single or two individuals, and their resulting trajectories were filtered to be smoothed. In the case of single fish tracking, we first applied a median filter (*n_win_* = 8 for position, *n_win_* = 15 for orientation) to exclude occasional outliers resulting from excess noise, and applied a triangular filter (*n_win_* = 15 for position, *n_win_* = 30 for orientation) to smooth the traces. The orientation traces were unwrapped before applying a filter to prevent jump-associated artifacts. In the case of dyads tracking, we first separated the traces of two individuals before applying the filters. An EOD pulse was associated with an individual by using its previously identified location having the closest position and orientation. Occasionally, the EOD pulses from two individuals temporally overlapped and produced collided pulses; this resulted in a lower tracking accuracy, since the collided pulses poorly matched the RSIs predicted by the 2D ideal dipole model. Hence, the collided pulses were detected by using their dot-product values as an exclusion criterion, and values below 0.9 were removed from the tracked trajectory.

### Visual Tracking Method

Our dipole tracking results were compared with automated visual tracking to quantify the accuracy of our dipole localization. We designed a visual tracking algorithm based on Windsor et al. [21]. Since fish appeared darker than the background, the background image was subtracted from the recorded images to obtain isolated images of the fish. Binary images were generated by applying an intensity threshold, and the largest blob was chosen after removing speckles by using an image dilation operation. The center of mass position and the orientation of the blob were found using the *regionprop* function provided by the Matlab image processing toolbox. The tail-bending angle was visually determined from the angle between two lines formed by three feature points at the head, the center of mass, and the tail. The head and the tail points were determined at the two end points of the midline, which was extracted from the blob using an image skeletonization function [58]. We determined a correct head orientation by manually assigning the head orientation for the first frame, and the head orientations for the subsequent frames were automatically determined using the previously determined values. The fish’s orientation returned by the *regionprop* function was compared with the corrected orientation from the previous frame, and the new orientation was flipped if the angular difference exceeded 90°. In the case of fish dyads tracking, we separately tracked each individual by initially defining a region of interest (ROI) around the fish being tracked, and the ROI locations were updated in subsequent frames by tracking the center of mass. The ROI served to exclude the other fish’s image when they were far apart. If the other fish’s image partly appeared in the ROI, it was automatically removed by detecting blobs touching the ROI boundary. In rare cases, the blobs of two fish merged when they made a direct contact, and we manually deleted the other fish’s image by drawing a polygon mask.

### Quantification of the Dipole Tracking Accuracy

Our dipole localization accuracy was quantified by comparing the position and orientation of an estimated dipole location with the visual tracking. The position errors were quantified by measuring the distance from the estimated dipole position to the center of mass point of the image. Similarly, the orientation errors were quantified by taking the absolute difference between the estimated dipole orientation and the orientation of the major elliptical axis from the image blob analysis. The position and orientation errors were quantified using the dipole locations estimated from the earlier part of the pulse phase (225 µsec before the reference timing), when the current source is mostly concentrated at the center of the body. The dipole locations estimated from each EOD pulse were smoothed and resampled at the image capture times (15 FPS). The number of samples for the dipole tracking error statistics (*n*) corresponds to the number of image frames used to compute the error. The tail-bending angle errors were quantified by taking the difference between the values determined from the two estimated dipole orientation (the central and the tail regions), and the values determined from the image analysis.

## Results

We applied our dipole localization algorithm to track freely swimming electric fish (20∼24 cm in length) in a shallow and circular body of water (10±2 cm depth, 1.5 m diameter). The dipole tracking accuracy was validated by an automated visual tracking protocol, and the positions and orientations determined by the two methods closely agreed (90% within 5 cm or 13°) under various test conditions. Our dipole tracking method was accurate and reliable at locations near the tank boundary or an object, and the error remained small when fish bent its tail during turning movements. Our method can therefore be used to estimate the distance between any part of the fish (e.g. its head) and an object (e.g. a landmark or prey). The tail-bending angles were determined by comparing the orientations of two dipoles at the central and tail regions (see Methods), and they correlated well (*ρ_corr = _*0.7631) with the visual observations. Our method was applied to track a fish pair or “dyads”, and the localization error remained similar to that for single fish tracking. EOD pulses produced by different individuals were identified by their dipole source locations, such that the individual identities could be reliably associated with their tracking. Our method can therefore also serve to relate the distances between two fish (e.g. between heads, tails, or head and tail) and the electric communication signals they emit [59].

### Single Fish Tracking Accuracy

**Figure 5:**
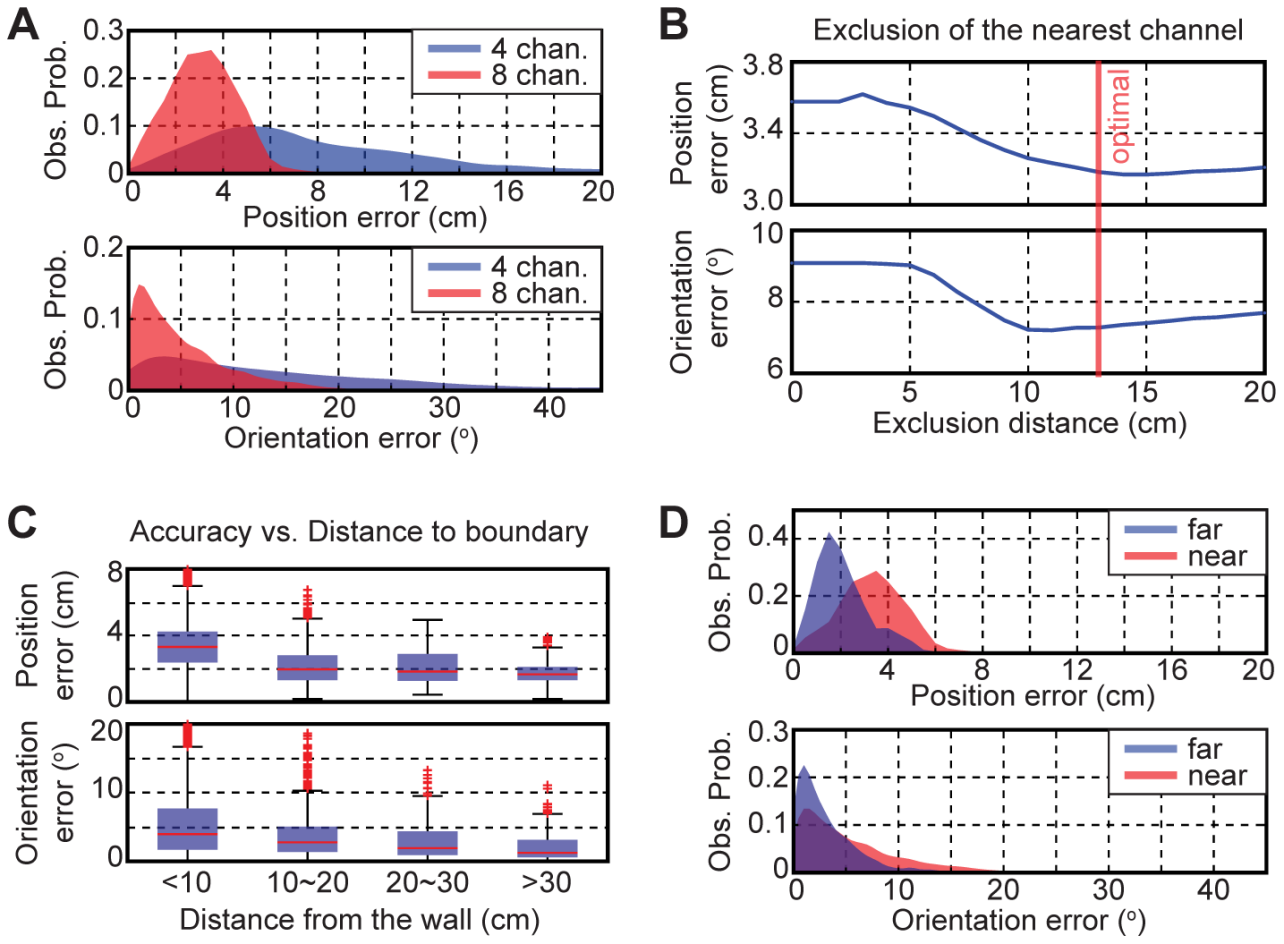
Single fish tracking accuracy. **A**) The error distribution of the dipole tracking for the four (blue) and eight (red) channel configurations (*Obs. Prob.*: Observation probability). **B**) The tracking accuracy improved after excluding the channel nearest to the fish within a set exclusion distance. **C**) The tracking errors were plotted vs. the distance from the tank wall. On each box, the central mark (red line) is the median, the edges of the box are the 25th and 75th percentiles, and the whiskers extend to the most extreme data points not considered outliers. Outliers are plotted individually (red markers): a value that is more than 1.5 times the interquartile range away from the top or bottom of the box as determined using the *boxplot* function (Statistics toolbox for Matlab). **D**) The error distributions of the dipole tracking near (<10 cm, red) and far (>10 cm, blue) from the tank wall.

We applied our dipole-tracking algorithm to track single freely swimming fish in a shallow and featureless tank, and the tracking accuracy was quantified using a visual tracking algorithm (see Methods). Figure 5A compares the tracking errors between the four (*4P90*) and the eight channel (*8P67.5*) configurations. The average errors of the eight-channel configuration (2.5±1.2 cm, 5.0±4.8°, *n* = 10^4^) were significantly lower than the four-channel case (9.4±9.1 cm, 15.7±14.8°, *n* = 10^4^) as expected from the simulations (Fig. 5A, Table 2). The position and orientation errors were significantly reduced after excluding a channel if its electrode was within a set exclusion distance from the fish’s center of the body (Fig. 5B). The position and orientation errors were minimized at the exclusion distance of 13 cm. The tracking accuracy degraded as animals approached the tank wall (Fig. 5C). 90% of all errors fell within 5.3 cm or 13.1° (*n_samples_* = 2×10^4^, *n_animals_* = 2) at all locations (Table 2). Figure 4D compares the error distributions between the locations near (<10 cm) and far (≥10 cm) from the wall; each fish was observed for 666.7 s to compute this distribution. The average tracking errors (mean ± SD) at the near locations were 1.4 cm and 3.7° higher than the errors at the far locations (2.2±1.1 cm, 2.7±2.4°, *n* = 1802). A short video of a single fish tracking is available in the (Video S1). In summary, our dipole tracking algorithm could accurately track single freely swimming electric fish at most locations within the tank using only eight pairs of recording electrodes, with the electrodes in each pair being at an angle of 67.5°.

**Table 2:** Summary of the tracking accuracy under different test conditions.

Test			Position Error (cm)			Orientation Error (°)		
*Category*	*conditions*	*n*	*mean*	*SD*	*Q90*	*mean*	*SD*	*Q90*
Boundary Dist.^*1^	far (≥10 cm)	1802	2.2	1.1	3.7	2.7	2.4	5.8
	near (<10 cm)	17995	3.6	1.4	5.4	6.4	6.1	13.5
Object Dist.^*2^	far (≥20 cm)	172	1.4	0.8	2.6	2.7	2.4	5.9
	near (<20 cm)	110	3.0	1.4	4.6	3.7	3.8	9.4
Tail Bending ^*1^	small (<10°)	1802	2.2	1.1	3.7	2.7	2.4	5.8
	large (≥10°)	203	2.5	1.0	3.8	7.0	4.5	13.1
Social tracking ^*1^	single	20000	3.5	1.5	5.3	6.0	5.9	13.1
	dyad	20000	3.6	1.4	5.4	6.0	6.6	12.9
No. Channels ^*2^	4 chan.	10000	9.4	9.1	17.1	15.7	14.8	34.8
	8 chan.	10000	2.5	1.2	4.1	5.0	4.8	11.5
Fish size (far) ^*3^	Large (31 g)	1377	2.1	1.1	3.8	3.1	3.0	6.9
	Small (20 g)	628	2.5	0.9	3.7	3.2	2.9	7.1
Fish size (all) ^*4^	Large (31 g)	10000	3.2	1.4	5.0	5.1	5.0	11.7
	Small (20 g)	10000	3.8	1.4	5.6	6.9	6.6	14.2

The dipole tracking errors under different test conditions are summarized in this table.

*1: Data pooled from all animals.

*2: Data from larger fish.

*3: Far from the wall (>10 cm).

*4: All locations.

### Effects of an Object

**Figure 6:**
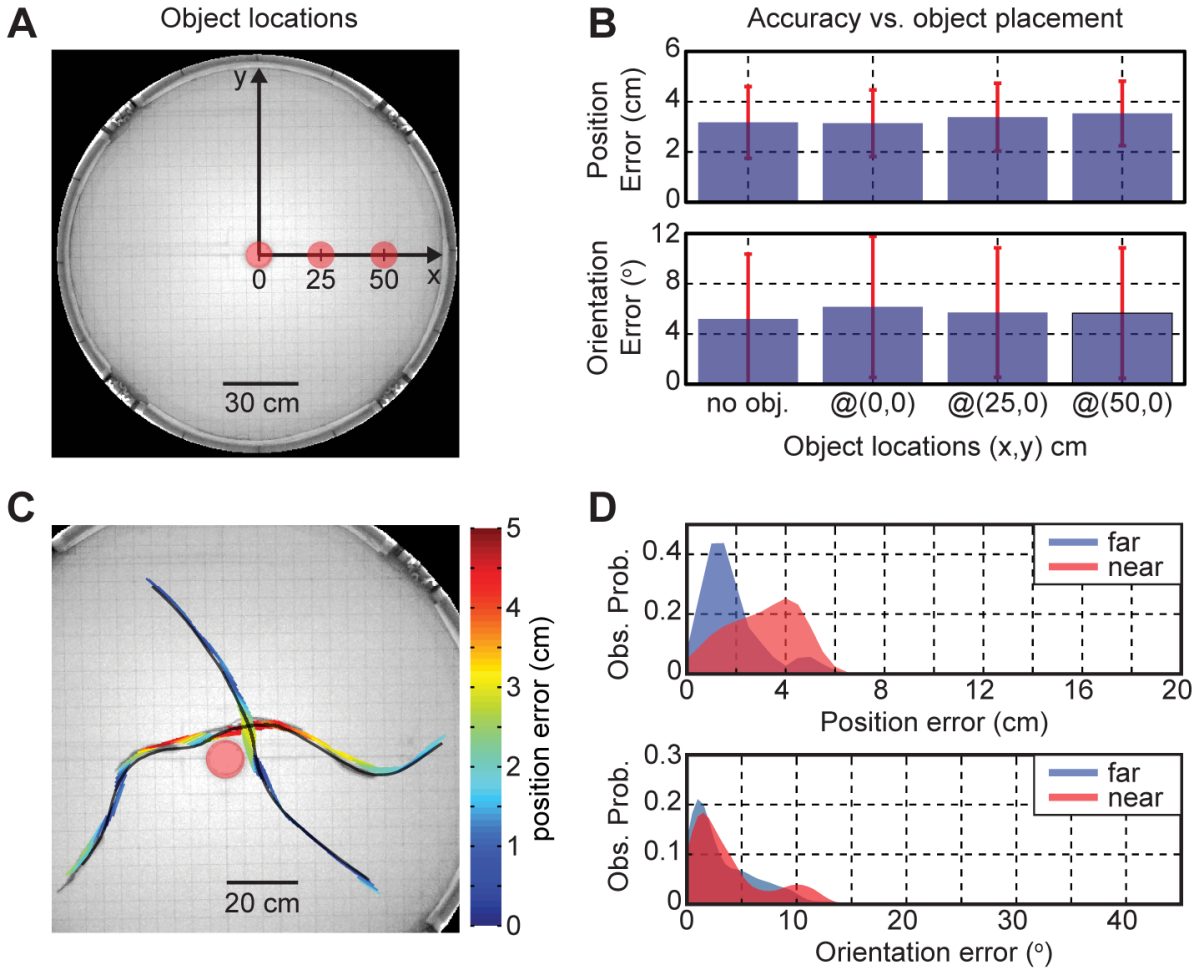
Effects of an object on the dipole tracking accuracy. **A**) The three object locations tested are marked in red circles, and our coordinates system is shown. **B**) The dipole tracking errors for the three object locations and the control (*no obj.*: no object placed) averaged over all locations visited by fish for 666.7 sec. **C**) A false color representation of the dipole tracking errors when fish passes by an object. The visually determined traces are shown in grey, and the object is marked as a red circle. **D**) The error distributions of the dipole tracking near (<20 cm, red) and far (>20 cm, blue) from the object’s surface.

We studied effects of a dielectric object on the dipole localization accuracy by placing a cylindrical plastic object (10 cm diameter, 15 cm height) at different locations (Fig. 6A). In theory, the object placed in water will distort the electric field and increase differences between the measured and predicted values; but according to our test, the localization errors were not affected by the object placed at three different locations (Fig. 6B). Figure 6C illustrates trajectories of a fish when it closely passed by the object, and the false colors represent the position error. The fish images were superimposed every 1 sec interval, and a movie version of Figure 6C is available in the (Video S2). The electrically tracked traces (color-coded) closely agreed (<5 cm) with the visually tracked traces (shown in grey) near the object. Figure 6D shows the error distributions when the fish was near (<20 cm) or far (>20 cm) from the object’s surface while the object was placed at the center of the tank. In order to examine the effect of an object alone, the locations near the wall (<10 cm) and when the tail-bending angles exceeded 10° were excluded from our analysis. The mean tracking errors when the fish was near the object were 3.0±1.4 cm and 3.7±3.8° (90% within 4.6 cm or 9.4°, *n* = 110); and the tracking errors when the fish was far from the object were 1.4±0.8 cm and 2.7±2.4° (90% within 2.6 cm or 5.9°, *n* = 172) (Table 2). In summary, the presence of an object in water did not significantly decrease the dipole tracking accuracy even when the fish closely passed by the object. Our method permits us to determine the EOD pulse rate as a function of the distance from a fish to an object, which will be relevant for studies such as Pereira et al. [60] and Hofmann et al. [61].

### Effects of Tail Bending

**Figure 7:**
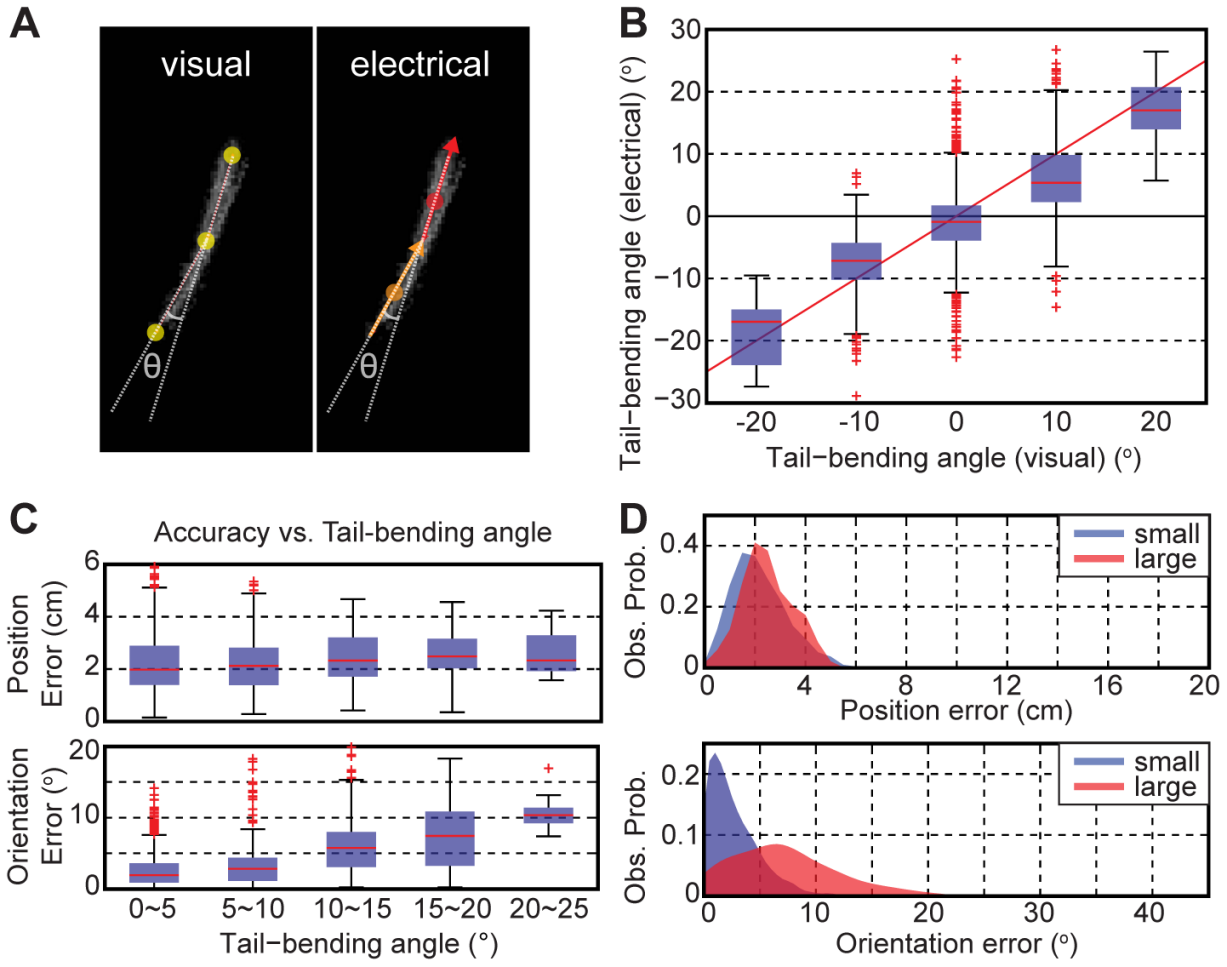
Effects of tail bending on the dipole tracking accuracy. **A**) Tail-bending angles were visually determined (left) using three feature points (head, tail, and center of the body), and also electrically determined using two dipoles at the central and one in the tail regions (right). **B**) The electrically determined tail-bending angles correlated well (*ρ_corr_* = 0.7631) with the visually determined values. **C**) The dipole tracking errors plotted as a function of the tail-bending angles. **D**) The error distributions of the dipole tracking for the small (<10°) and large (>10°) tail-bending angles. The locations near the tank wall (<10 cm) were excluded from our analysis to remove the near-field effect.

We quantified the dipole tracking errors during fish’s tail-bending behavior using visual recordings. The tail-bending angle was determined using three feature points (center of mass, head, and tail ends) from an image; and it was also electrically determined from two dipole orientations at the central and the tail regions (Fig. 7A, see Methods). Figure 7B shows a linear correlation between the electrically and the visually determined tail-bending angles (*ρ_corr_* = 0.7631, *n_samples_* = 2005, *n_animals_* = 2). Locations near the wall (<10 cm) were excluded from our analysis due to inaccurate visual tracking near the wall. The absolute tail-bending error was 3.7±3.9°, and 90% of all errors were within 7.9°. The orientation error increased linearly with the tail-bending angle, but the position error was not significantly affected by the tail bending (Fig. 7C). Figure 7D compares the distributions of the tracking errors during small (<10°) versus large (>10°) tail-bending bouts. The mean orientation error increased by 4.3° during the large tail-bending bouts relative to the small tail-bending bouts, while the mean position error increased only by 0.3 cm. In summary, the tail-bending angle could be determined from the electrical measurements alone, and the dipole tracking errors remained reasonably small during large tail bending (90% of errors within 5.8 cm or 13.8°). It has been hypothesized that the tail bending may be used by WEF for active sensing of objects in their surroundings [61]–[64]; our method will thus permit direct tests of this hypothesis.

### Fish Dyads Tracking

**Figure 8:**
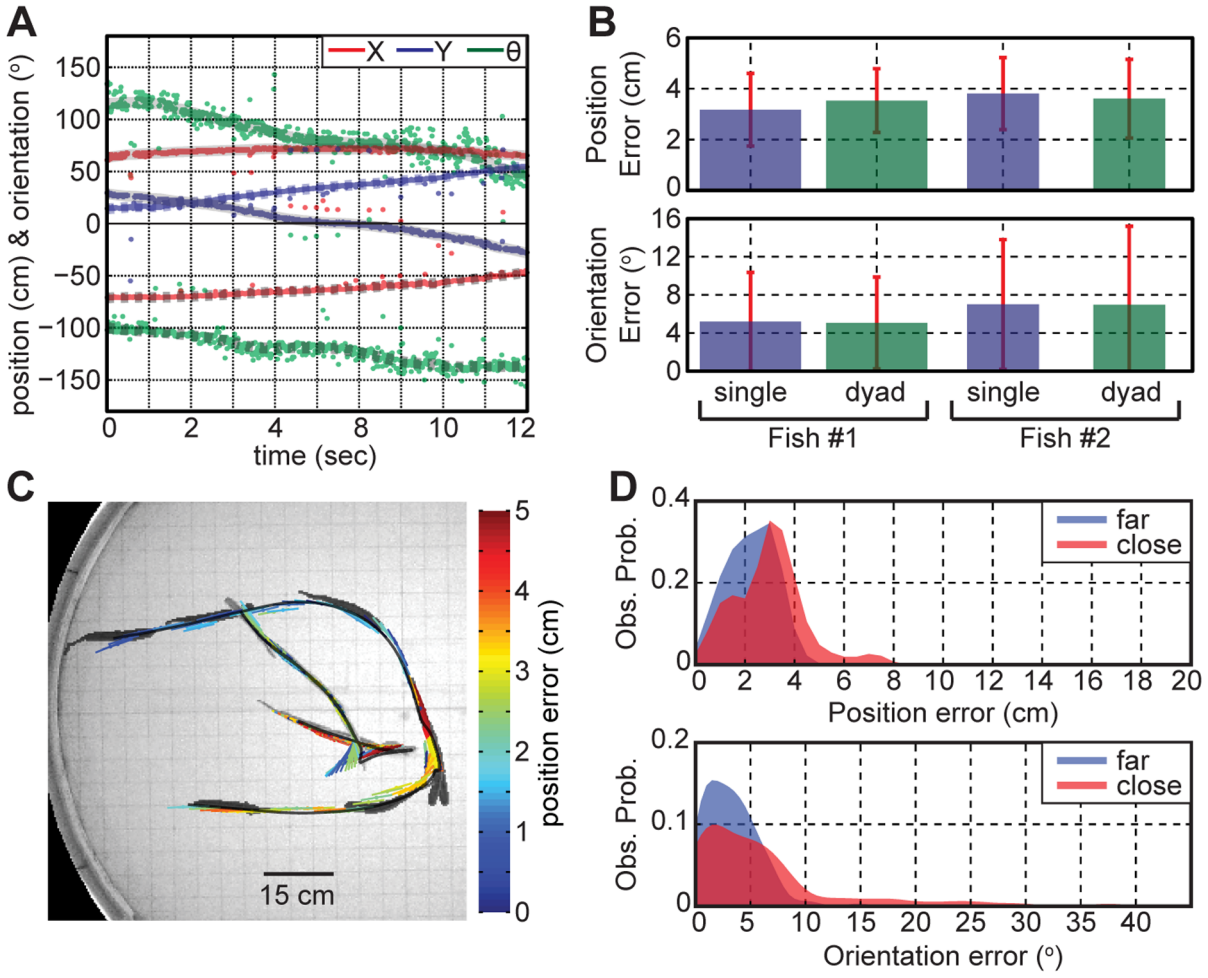
Fish dyads tracking accuracy. **A**) The traces of two fish were separated after the dipole localization. **B**) Comparison of the tracking errors between the single and dyads tracking for each fish. **C**) A false color representation of the tracking errors during close encounter between animals. Fish images were superimposed every 1 sec interval, and the larger fish is shown darker (Fish #1). **D**) The error distributions of the dipole tracking at a close (<40 cm) and far (>40 cm) distance between fish. The distances were measured to the center of the body, and the locations near the tank wall (<10 cm) were excluded from our analysis to remove the near-field effect.

Our dipole tracking system was adapted to track fish dyads both accurately and reliably. First, the dipole localization was carried out on mixed EOD pulses, and the tracked locations were individually identified on the basis of the nearest neighbors (see Methods). The EOD pulses occasionally collided when two fish emitted EOD pulses nearly simultaneously. Nevertheless, the LUT matching scores (dot product values) permitted detection and removal of the collided pulses from the traces (see the outliers of the traces in Fig. 8A). The dipole tracking errors of each individual were compared between the single and the dyads tracking (Fig. 8B), and the tracking errors were similar in both cases. The larger fish (animal #1, 24.0±0.5 cm, 31±1 g) had 0.6 cm and 1.8° less mean tracking errors than the smaller fish (animal #2, 20.5±0.5 cm, 20±1 g), since the visual tracking accuracy was higher for the larger fish near the wall in particular. Figure 8C illustrates the tracking errors during a close encounter between two individuals, and the electrically tracked traces (color-coded) closely agreed (<5 cm) with the visually tracked traces. The fish images were superimposed every 1 sec interval, and the larger fish is shown darker. A movie version of Figure 8C is available in the (Video S3). The distributions of the tracking errors when two fish were closer (<40 cm) or further apart (>40 cm) are shown in Figure 8D. The mean tracking errors when the fish dyads were near were 3.0±1.4 cm and 6.3±7.6° (90% within 4.4 cm or 16.7°, *n* = 186); and the errors when they were far apart were 2.2±0.9 cm and 3.2±2.4° (90% within 3.3 cm or 6.6°, *n* = 142). In summary, our dipole tracking method could simultaneously track fish dyads accurately even during close encounters. Since we time each EOD pulse of each fish to estimate their locations, our method permits determination of communication-associated EOD pulse patterning as a function of distance between the fish, which will be relevant to studies such as Hupé et al. [59] and Yu et al. [16].

## Discussion

### Significance of Our Paper

In this paper, we introduced a practical real-time electrical tracking system to locate relatively slowly moving dipole sources in a shallow homogeneous medium. Our dipole tracking method was originally developed to study naturalistic animal behaviors where visual observation is difficult to quantify due to the presence of objects or other animals. Our unique approach to the inverse problem was to solve forward problems at many possible locations, and to find a matching solution from a LUT constructed from the ideal dipole model. The forward problems were solved once, stored, and reused multiple times to increase the subsequent search speed. The dipole search operation could be performed in parallel by multiple processors or GPU if the LUT size becomes larger, or when faster search speed is required. In our studies, we tracked three location parameters (*x, y, θ*) in 2D, but the number of location parameters could be increased to locate dipoles in 3D. The 3D tracking requires at least five location parameters (*x, y, z, θ_azimuth_, θ_elevation_*) to be determined, thus this would require a greater number of recording channels and computational time. Unlike some other electrical tracking methods, our dipole tracking method does not require prior individual specific calibrations, since the dipole search algorithm relies on the relative RSI values between channels. This calibration-free approach of our tracking method would be particularly useful for a field recording setting, where a prior individual-specific calibration is difficult or impossible [49]. Although we used a circular tank, a tank shape is not critical to achieve high tracking accuracy as long as all electrodes are positioned on the tank wall. In the case of the square grid layout, high tracking accuracy could be achieved if the electrodes are sufficiently away from the boundary. The tracking accuracy did not noticeably increase when an object was placed in the tank, but placing too many or too large objects would deteriorate the tracking accuracy, more so if the conductivity differences with the surrounding water were large.

### Limitations of Visual Tracking

Visual tracking provides direct measurements of animal’s location, and it is the most widely used method of tracking animals. Automated visual tracking relies on computer vision algorithms to infer an animal’s location, but visual algorithms react sensitively to changes in lighting conditions of the environment such as shadows, reflections, and glares [65]. Imaging through water introduces additional challenges to visual tracking algorithms such as: glares produced on the water surface when light illumination is projected from the top, ripples triggered by animal’s movement, absorption of the infrared spectrum while observing nocturnal animals, and animals’ reflections on tank walls. To address these issues, homogenous light illumination could be projected from below a transparent tank to produce high contrast images [21]. However, this solution requires an aquarium to be raised above a light source; thus it is inadequate for a very large aquarium, or if heating is required from underneath the aquarium for tropical fish. Determining an animal’s posture such as a tail-bending angle could become a challenge when imaging a small animal in a large environment, due to the limited number of pixels available from an animal. Multiple cameras could be used to track animals in a large or a visually complex environment, but image frames must be synchronized and perspective angles of all cameras need to be calibrated to combine images from multiple cameras. Video recordings generally produce large amounts of data, thus recording durations are limited by available data storage.

Quantitative study of social interactions is an active area of study in behavioral biology, and the spatial aspect of social interactions provides valuable insights into animal communication [37]. Automated visual tracking could be applied to study multiple animals; but the visual algorithm must handle special cases such as when two animals overlap, and still maintain reliable individual identification. Visual markers [17]–[19] or naturally occurring stripe patterns [66] could be used to identify different individuals. However, fluorescent tags may not be visible under infrared illumination or invasive to use in animals, and the stripe patterns may be absent in some species. A model-based visual tracking method was proposed by Ibrahim et al. [22] to dissociate closely interacting *c. elegans* and zebrafish. Also, mesh models were fitted to images of closely interacting animals by maximizing statistical likelihood using past movement trajectories (Kalman filter); but this tracking method requires high-resolution images of animals and is more computationally demanding.

### Advantages of Electrical Tracking

Electrical tracking of WEF may be more appropriate in circumstances where visual tracking offers limited reliability or accuracy. One of the most significant differences between visual and electrical tracking is that the latter does not require the line of sight between a source and a detector. In naturalistic settings, animals generally prefer to hide, or they often contact other animals during social interactions. In such cases, electrical tracking could provide high reliability and accuracy. In field studies, radar telemetry is widely used to track insects carrying passive radio tags, which reflect microwave signals at their characteristic frequencies [26], [27]. More recently, RFID tags were used to study social behaviors of multiple rats in an enriched environment [7], [25]. However, radar telemetry offers limited detection range in an aquatic environment since water attenuates the radio frequency (RF) spectrum, especially when the conductivity of the surrounding water is high. Also, radio tags can impede with an animals’ mobility, or may require invasive surgical implantation of tags.

In contrast, our dipole tracking method makes use of an animal’s self-generated electrical signal, thus no tracking tags are required to track or discriminate between individuals. Our method is practically suited for an aquatic environment since the animal’s electric field can be detected using a simple setup of inexpensive electrodes, and the lower frequency band (<10 KHz) of physiological signals travel further in water than the RF spectrum used in radio telemetry. The detection range or accuracy could be enhanced by adding more recording dipole electrodes. It is much simpler to combine signals from multiple electrodes than combining 2D images from multiple cameras. Due to lower data storage demand, long-term behavioral monitoring becomes possible for capturing rarely occurring behaviors. Furthermore, poor visibility conditions in murky water do not affect our electrical tracking accuracy. Our method can also locate an animal obstructed by a shelter, as long as the shelter is electrically transparent such as a porous clay pot or plastic tubing with holes.

### Areas to Improve

The accuracy of our dipole localization decreased near the tank wall or during the tail bending bouts. When fish was very close to the tank wall, it occasionally contacted one of the recording electrodes and caused amplifier saturation. These occasional amplifier saturations compromised the dipole tracking accuracy near the wall. To improve the accuracy near the wall, the dipole model of the WEF could include the effects of the physically distributed current sources [11], [67]. This model could explain the near field effect better, but a calibration of the current source distribution would be required [67] for each individual from the visual and electrical measurements. During our dipole tracking, larger tail-bending bouts increased the orientation error because the visual tracking weighs the orientation of the head region more due to its larger area, whereas our dipole tracking weighs more toward the central region (Fig. 7B). In order to quantify the dipole tracking errors more accurately, the visual tracking could be performed using infrared-reflective tags. These tags could be sutured or glued on fish’s body to serve as visual markers for precisely measuring the position, orientation, and the tail-bending angle of the fish. The reflective markers would produce high contrast images and improve the visual tracking accuracy near the tank wall in particular, where shadows and reflections often decrease the tracking accuracy.

#### Combining the visual and the electrical tracking

In some cases, the EOD production of WEF is interrupted during social interactions or after a delivery of predatory stimuli [68], [69]. In *Gymnotus* species, the EOD can pause for several seconds during social interactions, or for up to a few minutes after threatening (predatory) stimuli. Our dipole tracking requires a continuous EOD production, but a WEF may drift away without emitting an EOD. The trajectory during a short EOD interruption may be reconstructed by extrapolating and joining the trajectories before and after the pause. WEF tend to be stationary during longer EOD pauses, but if they move, the movement trajectory could be determined from a concurrent video recording. Combination of concurrent video and electrical recordings will complement the reliability and accuracy of each tracking method. Our dipole tracking can separate different individuals during close social encounters, or locate animals when obstructed from view more reliably than the visual tracking. The dipole tracking could increase the visual tracking speed if it was is used to define the region of interest in images, or it could direct the viewing angle of a camera in real time if a motorized control is available for monitoring a large area.

#### Tracking wave-type species

In contrast to the pulse-type species we studied, wave-type species generate their EOD in a continuous and sinusoidal manner. It would require only small modifications to track a single wave-type species. First, the EOD timing reference would need to be determined from the envelope of the rectified and summed waveforms, and the slopes at each channel would be measured at some optimal time separation from the reference timing. The waveform polarity could be determined from the rectified waveforms by using the asymmetry between the positive and negative phases of the waveform. In the case of social tracking, the signal source separation between different individuals of the wave-type species would be difficult in the time domain because the EODs from different individuals constantly interfere. Furthermore, the wave-type species exhibit jamming avoidance responses (JAR) in social settings by shifting their EOD frequencies [45], [46]. Therefore, the frequency domain would offer better signal separation between different individuals [49], and the RSIs could be measured after the signal separation for each individual.

### Possible Future Applications

#### Field studies and 3D tracking

Field studies can offer important new insights, since animals may exhibit different or novel behaviors in their natural settings. Using our dipole tracking method, data already collected from the field [49] could be re-analyzed to determine animals’ locations and movement trajectories. This spatial quantification could uncover individual foraging patterns, or social dynamics such as group sizes in their natural environments [70]. Since depth of water is difficult to control in field studies, our two-dimensional (2D) tracking developed for shallow water may need to be modified to include the vertical dimension. The 3D tracking may become necessary if the water depth becomes much greater than the height of fish, or when fish’s vertical motion is important for a study (e.g. air gulping or barrel rolls displays) [71]. The vertical profile electrodes used in our 2D tracking would not be optimal for the 3D tracking, but instead, a planar grid layout (Fig. 3A) would be more appropriate [49]. Similar to the 2D tracking, the 3D tracking would require the LUT to be constructed for each x, y, z positions and the azimuth and elevation angles. The elevation angle may not be critical for the 3D tracking although changes in the elevation angle lead to the changes in the dipole moment. Our tracking method is not affected by the changes in the absolute dipole moment since it uses the relative, normalized RSIs. The 3D visual tracking was performed in a lab setting by placing one camera above, and another camera in front of a transparent tank for studying prey capture behaviors in WEF [72]. However, this method may be difficult to apply in field settings mainly due to positioning of cameras and illumination sources underwater. In contrast, the 3D dipole tracking would be much simpler to deploy in the field settings for studying the behaviors of WEF in their natural habitats.

#### EOD amplitude measurement

Changes in EOD amplitude during circadian rhythm or during social encounters were reported by Markham et al. [73] in a different electric fish species (*Sternopygus macrurus*). In principle, our method could measure the EOD amplitude of freely swimming fish by comparing the ratio of the observed RSIs to the predicted RSIs in order to determine the dipole moment strength. To ensure close match between the predicted and the measured values, the RSI measurements need to be taken when a fish is sufficiently away from the electrodes, and when the tail-bending angle is small. In the studies conducted by Markham et al. [73], the EOD amplitude measurements were taken when a fish passed through a narrow tube situated between two compartments. Although their method allowed the EOD measurements from unrestrained fish over an extended period of time, the animals were required to spontaneously swim through the measurement tube. In comparison, our dipole tracking method would allow constant monitoring of the EOD amplitudes without requiring the animals to swim through the measurement tube.

## Supporting Information

Figure S1
**The method of image charges applied to the shallow body of water.** (**A**) The image currents of the current source *I* created by the top and the bottom dielectric interfaces are shown up to the two first order reflections *I_0_* and *I_1_*. (**B**) The net potential (*V_net_*) due to the current source and its image currents is plotted as a function of the number of reflections (*n_r_*). *V_net_* was measured at the electrode at a distance *r = d*, and normalized to 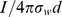 (*d*: depth of water). The current source was located at the height *d/2*. (**C**) The potential measured at the vertically oriented extended electrode was determined by averaging the potentials measured at different heights. (**D**) The numerically calculated potential of the vertically oriented electrode (*V_dip_*) is plotted in blue as a function of the normalized inverse distance (*R/d*)^−1^. The 2D ideal dipole voltage approximation is shown in red.(TIF)

Figure S2
**The method of image charges applied to the side circular boundary.** (**A**) The current source (*I*) and its image source (*I_1_*) are shown for the field location inside of the circular region. (**B**) Two image sources (*I_2_, I_3_*) are shown for the field location outside of the circular region. (**C**) The image current dipole (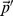) location is shown to calculate the differential potential between the electrodes pair (*e_1_*, *e_2_*).(TIF)

Text S1 **Text S2 derives the 2D ideal dipole approximation in shallow water from a three-dimensional electric field equation using a method of image charges.**
(DOC)

Video S1 **Video S1 illustrates dipole tracking results superimposed with an infrared video recording for a single WEF freely swimming in a shallow circular tank.**
(MP4)

Video S2 **Video S2 illustrates dipole tracking results superimposed with an infrared video recording, while a single WEF closely swims by a dielectric object placed at the center.**
(MP4)

Video S3 **Video S3 illustrates dipole tracking results superimposed with an infrared video recording for WEF dyads during their close encounter.**
(MP4)

Data S1 **Data S1 includes a software demonstration package for the dipole tracking with a sample dataset and an instruction manual.**
(ZIP)
