# Correction: Real-Time Localization of Moving Dipole Sources for Tracking Multiple Free-Swimming Weakly Electric Fish

**DOI:** 10.1371/journal.pone.0105293

**Published:** 2014-08-04

**Authors:** The PLOS ONE Staff

The equations in Text S1 do not display properly. Please view the correct Text S1 below.

## Supporting Information

Text S1 Text S1 derives the 2D ideal dipole approximation in shallow water from a three-dimensional electric field equation using a method of image charges.
(PDF)
