# Supplementary figures and images for: Real-Time Localization of Moving Dipole Sources for Tracking Multiple Free-Swimming Weakly Electric Fish
Source: PLoS One. 2013 Jun 21;8(6):e66596. doi: 10.1371/journal.pone.0066596 (PMC3689756; doi:10.1371/journal.pone.0066596)

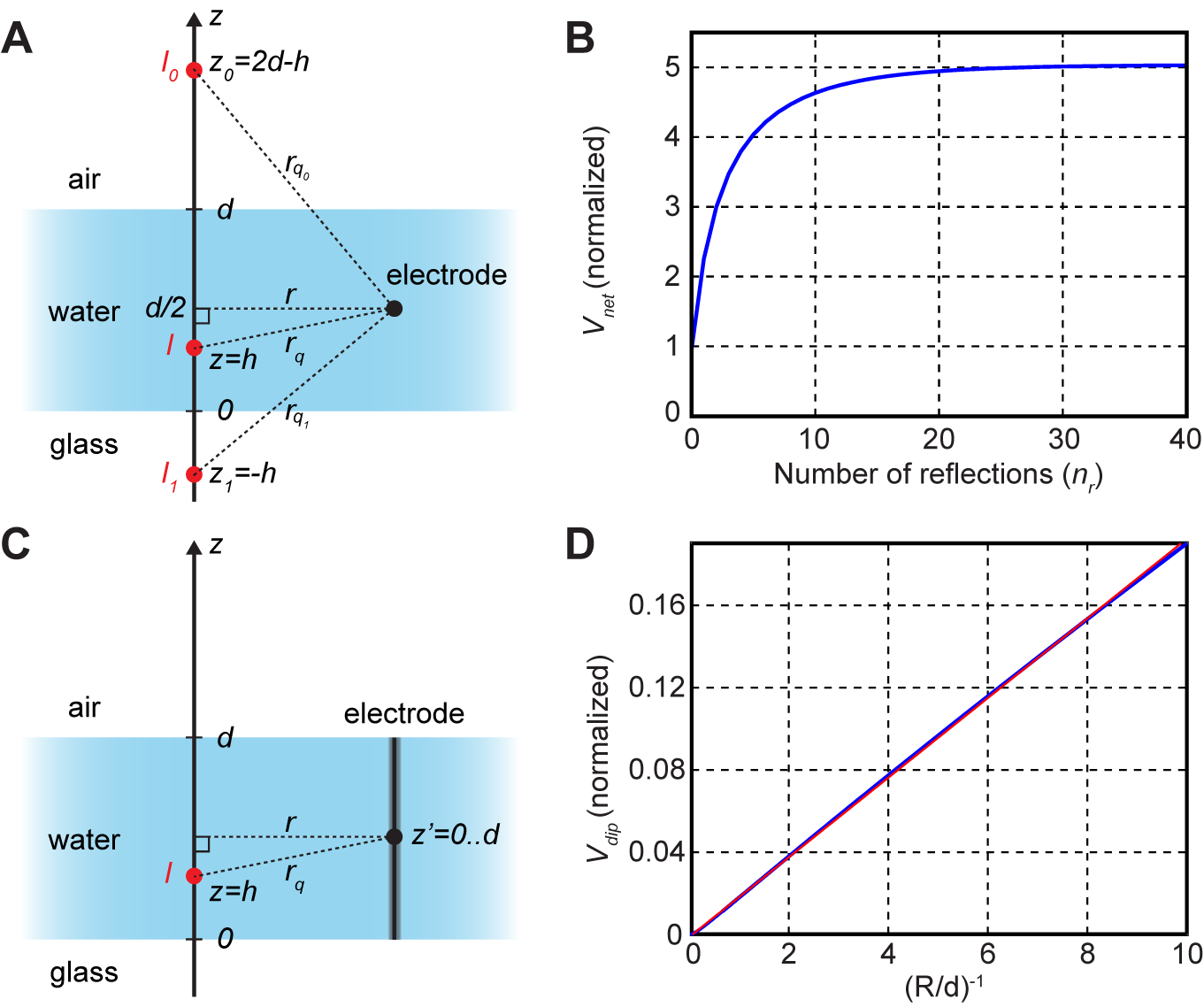

Supplement: Figure S1 — The method of image charges applied to the shallow body of water. (A) The image currents of the current source I created by the top and the bottom dielectric interfaces are shown up to the two first order reflections I0 and I1. (B) The net potential (Vnet) due to the current source and its image currents is plotted as a function of the number of reflections (nr). Vnet was measured at the electrode at a distance r = d, and normalized to (d: depth of water). The current source was located at the height d/2. (C) The potential measured at the vertically oriented extended electrode was determined by averaging the potentials measured at different heights. (D) The numerically calculated potential of the vertically oriented electrode (Vdip) is plotted in blue as a function of the normalized inverse distance (R/d)−1. The 2D ideal dipole voltage approximation is shown in red. (TIF) [file pone.0066596.s001.tif]

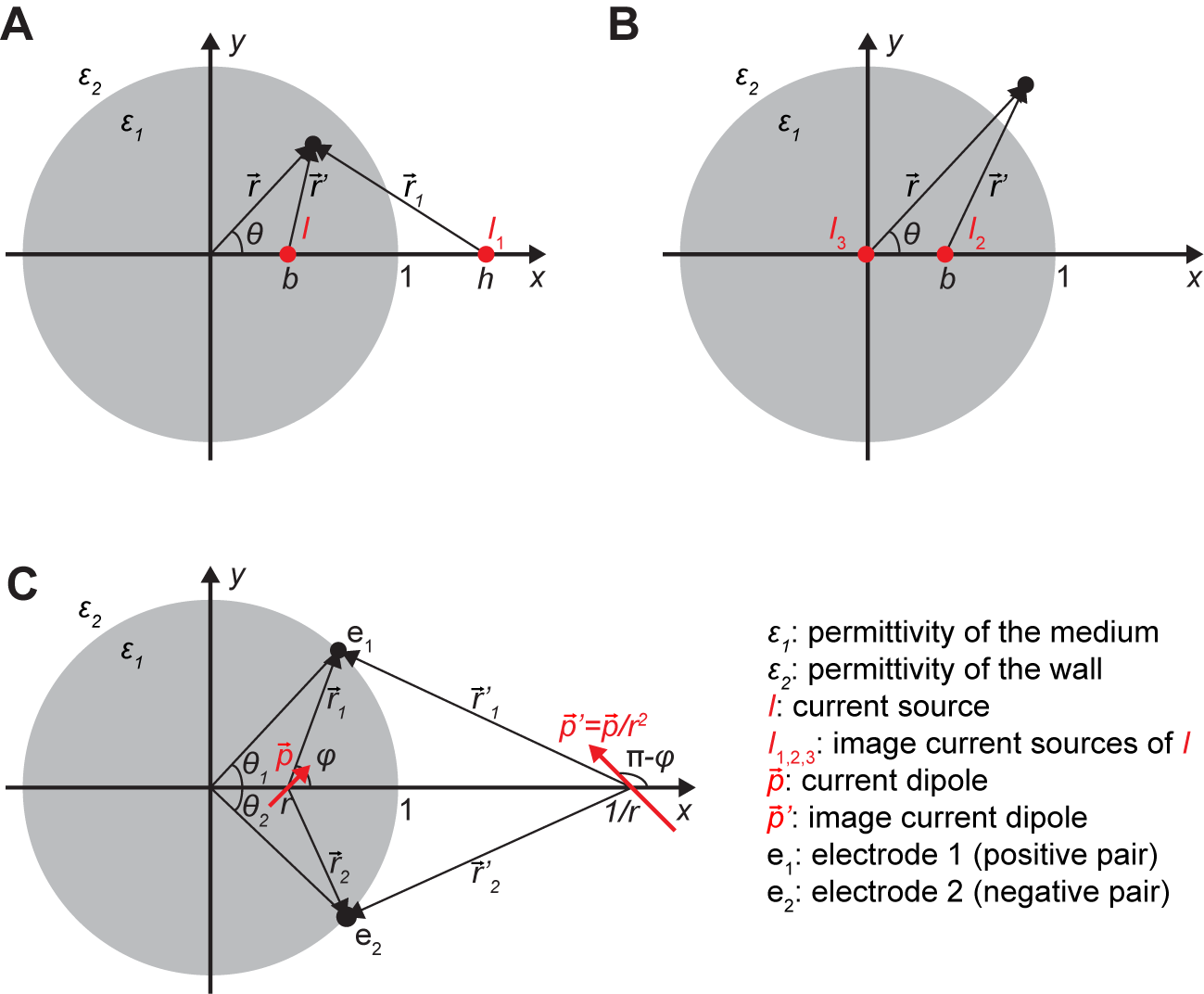

Supplement: Figure S2 — The method of image charges applied to the side circular boundary. (A) The current source (I) and its image source (I1) are shown for the field location inside of the circular region. (B) Two image sources (I2, I3) are shown for the field location outside of the circular region. (C) The image current dipole () location is shown to calculate the differential potential between the electrodes pair (e1, e2). (TIF) [file pone.0066596.s002.tif]
