# Supplementary material for: Real-Time Localization of Moving Dipole Sources for Tracking Multiple Free-Swimming Weakly Electric Fish
Source: PLoS One. 2013 Jun 21;8(6):e66596. doi: 10.1371/journal.pone.0066596 (PMC3689756; doi:10.1371/journal.pone.0066596)
Supplement: Text S1 — Text S2 derives the 2D ideal dipole approximation in shallow water from a three-dimensional electric field equation using a method of image charges. (DOC) [file pone.0066596.s003.doc]

**Supplementary Text S1**

# Validity of the 2D ideal dipole approximation in shallow water

In the main text, we used the two-dimensional ideal dipole formula to predict the voltage difference between a pair of electrodes. The 2D ideal dipole formula was derived by assuming an infinite 2D space without boundaries. In this text, we will derive a more realistic ideal dipole formula by including the depth dimension and the boundary conditions to show that the 3D ideal dipole formula reduces to the 2D ideal dipole formula for a shallow, circular body of water. Our analysis will include the effects of the boundaries and the finite electrode dimension, which influence the voltage difference between a pair of vertically oriented electrodes in the presence of a dipole. The dipole is situated in the circular body of water of a radius *R* and depth *d*; and the water is surround by the top, bottom, and side boundaries.

# 1. Effects of the top and bottom interfaces

First, let us consider the effects of the top and bottom interfaces on the electric fields, and assume an infinitely wide body of water of a depth *d*. The current dipole induces surface charges on the two dielectric interfaces, one on the top between the water and the air, and another at the bottom between the water and the glass. The induced surface charges contribute to the electrode potential, but it is difficult to directly determine the surface charge distributions. The method of image charges simplifies our analysis to determine the electrode potential, by replacing the surface charges with image current sources on the opposite side of each boundary [57,58]. Each dielectric boundary acts as a mirror surface to generate an image source *I’* on the opposite side of the boundary at an equal distance to the surface, having a magnitude:

$I'=\underbrace{\left(\frac{\varepsilon_{w}-\varepsilon_{k}}{\varepsilon_{w}+\varepsilon_{k}}\right)}_{\equiv\alpha_{k}}I=\alpha_{k}I,$ (S1.1)

where $\varepsilon_{w}$ is the permittivity of water, $\varepsilon_{k}$ is the permittivity of the neighboring media (air or glass), and $\alpha$is the attenuation factor. Note that the image source expression does not depend on the conductivity of the media to calculate the electric field inside of the circular region [58]. In our problem, the two parallel surfaces at the top and at the bottom create an infinite number of image sources by creating an infinite number of reflections. Each reflection by a surface *k* creates a new image source with an attenuation factor $\alpha_{k}$. For instance, the first order reflection creates two image sources: *I_0_* above the top interface at $z_{0}=2d-h$, and *I_1_* below the bottom interface at $z_{1}=-h$ (see Fig. S1A).

$I_{0}=\underbrace{\frac{\varepsilon_{w}-\varepsilon_{0}}{\varepsilon_{w}+\varepsilon_{0}}}_{\equiv\alpha_{0}}I=\alpha_{0}I, I_{1}=\underbrace{\frac{\varepsilon_{w}-\varepsilon_{1}}{\varepsilon_{w}+\varepsilon_{1}}}_{\equiv\alpha_{1}}I=\alpha_{1}I.$ (S1.2)

where $\varepsilon_{0}$ is the permittivity of air, and $\varepsilon_{1}$ is the permittivity of glass. Similarly, the second order reflections create image sources of $I_{0}$ and $I_{1}$. For example, the bottom surface creates a second order image source $I_{01}$ from the first order image source $I_{1}$ at a height of $z_{01}$, where:

$\left\{\begin{matrix} I_{01}=\alpha_{1}I_{0}=\alpha_{1}\left(\alpha_{0}I\right)=\alpha_{0}\alpha_{1}I, \\ z_{01}=-z_{0}=h-2d. \end{matrix}\right.$ (S1.3)

We can describe the reflection operations for each surface recursively. The reflection operations for the top interface (air-water interface, *k=0*) are:

$\left\{\begin{matrix} I_{\left\{k\right\}0}=f_{0}\left(I_{k}\right)=\alpha_{0}I_{k}, \\ z_{\left\{k\right\}0}=g_{0}\left(z_{k}\right)=2d-z_{k}. \end{matrix}\right.$ (S1.4)

Note that the top surface will always reflect an image charge located below the bottom surface ($z_{k}<0$), such that the distance to the new image source ($z_{\left\{k\right\}0}$) from the original current source will always increase: . The reflection operations for the bottom interface (glass-water interface, *k=1*) are:

$\left\{\begin{matrix} I_{\left\{k\right\}1}=f_{1}\left(I_{k}\right)=\alpha_{1}I_{k}, \\ z_{\left\{k\right\}1}=g_{1}\left(z_{k}\right)=-z_{k}. \end{matrix}\right.$ (S1.5)

Now, we can express the potential at a field location $\vec{r}$due to an image source $I_{k}$:

$V\left(\vec{r}|I_{k}\right)=-\frac{cI_{k}}{\left|\vec{r}-\vec{r}_{k}\right|}=-\frac{cI_{k}}{\sqrt{r^{2}+\left(z-z_{k}\right)^{2}}},$ (S1.6)

where $c=\left(4\pi\sigma_{w}\right)^{-1}$ is the constant of proportionality, and $\sigma_{w}$ is the conductivity of water. The net potential at $\vec{r}$due to the current source *I*, and its first order reflections is:

$\begin{matrix} V_{net}\left(\vec{r}\right)=V\left(\vec{r}|I\right)+V\left(\vec{r}|I_{0}\right)+V\left(\vec{r}|I_{1}\right), \\ =-\frac{cI}{\left|\vec{r}-\vec{r}_{I}\right|}-\frac{cI_{0}}{\left|\vec{r}-\vec{r}_{I_{0}}\right|}-\frac{cI_{1}}{\left|\vec{r}-\vec{r}_{I_{1}}\right|}, \\ =-cI\left[\left(r^{2}+\left(z-\frac{d}{2}\right)^{2}\right)^{-1/2}+\alpha_{0}\left(r^{2}+\left(z_{0}-\frac{d}{2}\right)^{2}\right)^{-1/2}+\alpha_{1}\left(r^{2}+\left(z_{1}-\frac{d}{2}\right)^{2}\right)^{-1/2}\right]. \end{matrix}$ (S1.7)

The net potential up to the second order reflections is:

$V_{net}\left(\vec{r}\right)=V\left(\vec{r}|I\right)+\underbrace{\left[V\left(\vec{r}|I_{0}\right)+V\left(\vec{r}|I_{1}\right)\right]}_{\text{first order reflections}}+\underbrace{\left[V\left(\vec{r}|I_{01}\right)+V\left(\vec{r}|I_{10}\right)\right]}_{\text{second order reflections}}.$ (S1.8)

Figure S1A shows the net potential calculated up to the *n^th^* reflection as a function of *n­*. Our numerical calculation indicates that the net potential converges. The convergence of the potential is expected since each reflection produces a weaker image source at a further location from the electrode.

# 2. Finite electrode dimension

Now, let us consider the effect of a finite electrode dimension. The potential at the surface of an electrode is equal throughout its surface since the electrode is a conductor, and the electrode measures the average of potentials at different heights. This could be shown by extending the proof based on connecting two conducting spheres. Thus, the electrode potential can be determined by averaging the potentials at different heights *h*, where $0\leq h\leq d$:

$V_{e}\left(\vec{r}|I\right)=-\frac{1}{d}\int_{z'=0}^{d} \frac{cI}{\sqrt{r^{2}+\left(z'-h\right)^{2}}}dz'=-\frac{cI}{d}\left(\sinh^{-1}\left(\frac{d-h}{r}\right)+\sinh^{-1}\left(\frac{h}{r}\right)\right).$ (S2.1)

If the distance from the current source to the electrode (=*r*) is much greater than the depth of water (=*d*): $d\ll r$, we can apply a Taylor approximation up to the first order:

$\left\{\begin{matrix} \sinh^{-1}\left(\frac{h}{r}\right)\approx\frac{h}{r}, \left|\frac{h}{r}\right|\ll1, \\ \sinh^{-1}\left(\frac{d-h}{r}\right)\approx\frac{d-h}{r}, \left|\frac{d-h}{r}\right|\ll1. \end{matrix}\right.$ (S2.2)

Substitute (S2.1) into (S2.2) to obtain the potential of a rod-shaped electrode:

(S2.3)

Therefore, the potential of a vertically oriented extended electrode due to a single current source is approximately equal to the potential of a point-like electrode if the electrode is sufficiently far from the current source relative to the depth of water.

# 3. Derivation of the two-dimensional ideal dipole model

Let us derive an expression for the potential due to an ideal dipole in a two-dimensional space. According to Gauss’ law, the electric field strength due to a current source (*I*) in 2D is:

$\oint_{r'=r} E\left(r'\right)dr'=\frac{I}{\sigma}\Rightarrow E=\frac{I}{\sigma\left(2\pi r\right)}=\frac{cI}{r},$ (S3.1)

where *c* is a positive constant, $\sigma$ is the conductivity of the medium and *r* is the distance from the current source to the field location. We can obtain the electric potential (*V*) by integrating the electric field from Eq (S3.1):

$V\left(r\right)=-\int E\left(a\right)da=-cI\ln r.$ (S3.2)

The potential due to a dipole is equal to the sum of contributions from the positive and negative current sources:

$V_{dip}=V_{+}-V_{-}=-cI\left(\ln r_{+}-\ln r_{-}\right),$ (S3.3)

where $r_{+}$ is the distance between the positive source to the field location, and $r_{-}$ is the distance between the negative source to the field location. According to the cosine law, the distances from the sources to the field location are:

$r_{\pm}=r\sqrt{1\mp\frac{d}{r}\cos\theta+\left(\frac{d}{2r}\right)^{2}},$ (S3.4)

where $\theta$ is the angle between the vectors $\vec{r}$ and $\vec{d}$ (Fig. 1C). Taking the *ln* of Eq (S3.4):

$\ln r_{\pm}=\ln r+\frac{1}{2}\ln\left(1\mp\frac{d}{r}\cos\theta+\left(\frac{d}{2r}\right)^{2}\right),$ (S3.5)

and substituting Eq (S3.5) in Eq (S3.3):

$V_{dip}=\frac{cI}{2}\left[\ln\left(1+\frac{d}{r}\cos\theta+\left(\frac{d}{2r}\right)^{2}\right)-\ln\left(1-\frac{d}{r}\cos\theta+\left(\frac{d}{2r}\right)^{2}\right)\right].$ (S3.6)

In the limit of $d\ll r$, Eq (S3.6) can be approximated as:

$V_{dip}\approx\frac{cI}{2}\ln\left(\frac{1+\left(d/r\right)\cos\theta}{1-\left(d/r\right)\cos\theta}\right).$ (S3.7)

We can apply a Taylor expansion formula:

$\frac{1}{2}\ln\left(\frac{1+x}{1-x}\right)=x+\frac{x^{3}}{3}+\frac{x^{5}}{5}+\ldots, \left|x\right|<1,$ (S3.8)

to Eq (S3.7) by setting $x\to\left(d/r\right)\cos\theta$, since $\left|\left(d/r\right)\cos\theta\right|<1$ from $d\ll r$. Applying Eq (S3.8) to Eq (S3.7) yields the ideal dipole potential in the two-dimensional case:

(S3.15)

where *p* is the current dipole moment.

Now, let us compare the potentials due to a current dipole placed in shallow water determined from the two-dimensional ideal dipole model approximation (S3.15) with numerical solutions. The dipole potential can be numerically calculated by estimating a dipole as a pair of closely spaced current source and sink, and summing all potential contributions due to the current sources and their image currents up to the 1000^th^ order. Figure S2D shows the potential of a vertically oriented electrode due to a current dipole as a function of the normalized inverse distance. The two current sources were separated by 0.01*d* at a depth of *z = d/2*, and oriented toward the electrode. Our numerical result (blue curve in Fig. S2D) confirms that the 2D ideal dipole voltage approximation (red line in Fig. S2) is valid for the potential of a vertically oriented extended electrode in shallow water. The 2D ideal dipole voltage approximation worked very well even when the dipole was located very close to the electrode (*r*~0.1*d*).

# 4. Effect of the circular boundary

A current dipole accumulates a surface charge on the interface between the circular plastic wall and the water, thus we also need to consider the effect of the circular boundary on our measurements. Let us treat our tank as a circle in a two-dimensional space, and use the method of image charges since the 2D approximation is shown to work well in the previous section. Let us consider a current source *I* oriented inside of the circular region. The inside region of the circular boundary has a conductivity of $\sigma_{1}$ and a permittivity of $\varepsilon_{1}$; and the outside region has a conductivity of $\sigma_{2}$ and a permittivity of $\varepsilon_{2}$. The aim is to find the magnitudes and the locations of the image sources corresponding to *I*. Since image sources cannot be located where the field is evaluated, we must separately find the image sources inside and outside of the circular region. To simplify our derivation, the length unit was normalized to the radius of the aquarium, such that the radius of the aquarium was set to one.

**Case 1**: Field location inside of the circular region (*r<1*):

We must place an image source *I_1_* outside of the region to compute the potential inside of the circular region (see Fig. S2A). According to the cosine law, the distance from the current sources to the field point $\vec{r}$ is:

$r'=\sqrt{r^{2}+b^{2}-2rb\cos\theta}, r_{1}=\sqrt{r^{2}+h^{2}-2rh\cos\theta}.$ (S4.1)

From Eq (6) of the main text, the net potential due to the current source *I* and its image source *I_1_* is:

$V_{in}\left(\vec{r}\right)=-\frac{I}{2\pi\sigma_{1}}\ln\left(r'\right)-\frac{I_{1}}{2\pi\sigma_{1}}\ln\left(r_{1}\right).$ (S4.2)

**Case 2**. Field location outside of the circular region (*r* > 1):

We must place an image source *I_3_* and replace the original current source *I* with *I_2_* inside of the circular region [56] to compute the potential outside of the region (see Fig. S2B).

(S4.3)

The constant is required to make the voltage continuous at the circular boundary such that: $V_{in}(r=1)=V_{out}\left(r=1\right)$. Now the electric field must satisfy the boundary conditions below:

$\left\{\begin{matrix} E_{in}^{\parallel}=E_{out}^{\parallel}\Rightarrow\left.\frac{d}{d\theta}V_{in}\right|_{r=a}=\left.\frac{d}{d\theta}V_{out}\right|_{r=a}, \\ \varepsilon_{1}E_{in}^{\perp}=\varepsilon_{2}E_{out}^{\perp}\Rightarrow\varepsilon_{1}\left.\frac{d}{dr}V_{in}\right|_{r=a}=\varepsilon_{2}\left.\frac{d}{dr}V_{out}\right|_{r=a}. \end{matrix}\right.$ (S4.4)

First, let us find the parallel components of the electric field ($E^{\parallel}$ ). From (S4.2) and (S4.3):

$\left\{\begin{matrix} \left.\frac{dV_{in}}{d\theta}\right|_{r=a}=-\frac{1}{2\pi\sigma_{1}}\left[\frac{Ib\sin\theta}{b^{2}-2b\cos\theta+1}+\frac{I_{1}h\sin\theta}{h^{2}-2h\cos\theta+1}\right], \\ \left.\frac{dV_{out}}{d\theta}\right|_{r=a}=-\frac{1}{2\pi\sigma_{2}}\left[\frac{I_{2}b\sin\theta}{b^{2}-2b\cos\theta+1}\right]. \end{matrix}\right.$ (S4.5)

Equating the above two equations using (S4.4):

$\begin{matrix} \sigma_{2}\left[\frac{Ib\sin\theta}{b^{2}-2b\cos\theta+1}+\frac{I_{1}h\sin\theta}{h^{2}-2h\cos\theta+1}\right]=\sigma_{1}\left[\frac{I_{2}b\sin\theta}{b^{2}-2b\cos\theta+1}\right], \\ \Rightarrow\frac{\sigma_{2}Ib}{b^{2}-2b\cos\theta+1}+\frac{\sigma_{2}I_{1}h}{h^{2}-2h\cos\theta+1}=\frac{\sigma_{1}I_{2}b}{b^{2}-2b\cos\theta+1}. \end{matrix}$ (S4.6)

Rationalizing (S4.6) yields:

$\sigma_{2}Ib\left(h^{2}-2h\cos\theta+1\right)+\sigma_{2}I_{1}h\left(b^{2}-2b\cos\theta+1\right)=\sigma_{1}I_{2}b\left(h^{2}-2h\cos\theta+1\right).$ (S4.7)

Separating cosine dependent and independent terms:

$\left[\sigma_{2}b\left(h^{2}+1\right)I+\sigma_{2}h\left(b^{2}+1\right)I_{1}-\sigma_{1}b\left(h^{2}+1\right)I_{2}\right]+2bh\cos\theta\left[-\sigma_{2}I-\sigma_{2}I_{1}+\sigma_{1}I_{2}\right]=0.$ (S4.8)

The equation above must be satisfied for any choice of $\theta$, thus:

(S4.9)

Substituting (S4.9) into (S4.8):

$\begin{matrix} \sigma_{2}b\left(h^{2}+1\right)I+\sigma_{2}h\left(b^{2}+1\right)I_{1}-\sigma_{2}b\left(h^{2}+1\right)\left(I+I_{1}\right)=0, \\ \Rightarrow\sigma_{2}I_{1}\left(hb^{2}+h-bh^{2}-b\right)=0\Rightarrow b+\frac{1}{b}=h+\frac{1}{h}. \end{matrix}$ (S4.10)

The solution to (S4.10) can be found using the quadratic formula, yielding: $h=\left\{b,1/b\right\}$. However, image sources cannot be located in the same region of the real current source, thus:

(S4.12)

Now, let us find the perpendicular component of the electric field ($E^{\perp}$). From (S4.2) and (S4.3):

$\left\{\begin{matrix} \left.\varepsilon_{1}\frac{dV_{in}}{dr}\right|_{r=a}=\frac{\varepsilon_{1}}{2\pi\sigma_{1}}\left[\frac{I\left(b\cos\theta-1\right)}{b^{2}-2b\cos\theta+1}+\frac{I_{1}\left(h\cos\theta-1\right)}{h^{2}-2h\cos\theta+1}\right], \\ \left.\varepsilon_{2}\frac{dV_{out}}{dr}\right|_{r=a}=\frac{\varepsilon_{2}}{2\pi\sigma_{2}}\left[\frac{I_{2}\left(b\cos\theta-1\right)}{b^{2}-2b\cos\theta+1}-I_{3}\right]. \end{matrix}\right.$ (S4.13)

Equating the above equations according to (S4.4):

$\frac{\varepsilon_{1}}{\sigma_{1}}\left(\frac{I\left(b\cos\theta-1\right)}{b^{2}-2b\cos\theta+1}+\frac{I_{1}\left(h\cos\theta-1\right)}{h^{2}-2h\cos\theta+1}\right)=\frac{\varepsilon_{2}}{\sigma_{2}}\left(\frac{I_{2}\left(b\cos\theta-1\right)}{b^{2}-2b\cos\theta+1}-I_{3}\right).$ (S4.14)

Substituting (S4.12) into (S4.14):

$\begin{matrix} \sigma_{2}\varepsilon_{1}\left[\frac{I\left(b\cos\theta-1\right)}{b^{2}-2b\cos\theta+1}+\frac{I_{1}\left(b\cos\theta-b^{2}\right)}{b^{2}-2b\cos\theta+1}\right]=\sigma_{1}\varepsilon_{2}\left[\frac{I_{2}\left(b\cos\theta-1\right)}{b^{2}-2b\cos\theta+1}-I_{3}\right], \\ \Rightarrow\sigma_{2}\varepsilon_{1}I\left(b\cos\theta-1\right)+\sigma_{2}\varepsilon_{1}I_{1}\left(b\cos\theta-b^{2}\right)=\sigma_{1}\varepsilon_{2}I_{2}\left(b\cos\theta-1\right)-\sigma_{1}\varepsilon_{2}I_{3}\left(b^{2}-2b\cos\theta+1\right), \\ \Rightarrow\left[-\sigma_{2}\varepsilon_{1}I-\sigma_{2}\varepsilon_{1}b^{2}I_{1}+\sigma_{1}\varepsilon_{2}I_{2}+\sigma_{1}\varepsilon_{2}\left(b^{2}+1\right)I_{3}\right]+b\cos\theta\left[\sigma_{2}\varepsilon_{1}I+\sigma_{2}\varepsilon_{1}I_{1}-\sigma_{1}\varepsilon_{2}I_{2}-2\sigma_{1}\varepsilon_{2}I_{3}\right]=0. \end{matrix}$ (S4.15)

Similarly, the equation above must be satisfied for any choice of $\theta$, thus:

$\left\{\begin{matrix} \sigma_{2}\varepsilon_{1}\left(I+I_{1}\right)=\sigma_{1}\varepsilon_{2}\left(I_{2}+2I_{3}\right), \\ \sigma_{2}\varepsilon_{1}\left(I+b^{2}I_{1}\right)=\sigma_{1}\varepsilon_{2}\left(I_{2}+\left(b^{2}+1\right)I_{3}\right)=0, \end{matrix}\right. \Rightarrow\left\{\begin{matrix} I_{1}=\frac{\sigma_{1}\varepsilon_{2}}{\sigma_{2}\varepsilon_{1}}I_{3}, \\ I_{2}=\frac{\sigma_{2}\varepsilon_{1}}{\sigma_{1}\varepsilon_{2}}\left(I-I_{1}\right). \end{matrix}\right.$ (S4.16)

Substituting (S4.16) into (S4.9) yields all the image sources:

(S4.17)

Observe that if $\sigma_{1}\gg\sigma_{2}$,$I_{2}$and $I_{3}$ vanishes and we obtain zero potential outside of the circular region, as expected from a body of water surrounded by a circular plastic wall.

# 5. Differential voltage due to an image dipole

Previously, we have found the potential due to a current source in 2D with the circular boundary by using the method of image charges. We can apply our previous results to determine the potential due to an ideal current dipole in 2D with the circular boundary condition. The circular dielectric boundary creates an image current dipole outside of the region, and we need to determine the potential difference between two electrodes due to the ideal current dipole (see Fig. S2C). The potential due to a current dipole in 2D is:

(S5.1)

where is the unit vector of the dipole $\vec{p}$. From (S5.1), the voltage difference between the two electrodes $e_{1}$ and $e_{2}$ (see Fig. S2C) is:

(S5.2)

where $\left|\vec{r}_{1,2}\right|^{2}=1-2r\cos\theta_{1,2}+r^{2}$. Now, let us compute the potential difference due to the image dipole. The image dipole is created outside of the circular boundary due to the reflection from the dielectric circular wall, and it has a magnitude of [56]:

$p'=\left(\frac{\varepsilon_{w}-\varepsilon_{p}}{\varepsilon_{w}+\varepsilon_{p}}\frac{1}{r^{2}}\right)p,$ (S5.3)

with the unit vector . The voltage difference due to the image dipole is:

(S5.4)

where $\left|\vec{r}_{1,2}^{'}\right|^{2}=1-2\cos\theta_{1,2}/r+1/r^{2}=\left|\vec{r}_{1,2}\right|^{2}/r^{2}$. It can be shown that $\Delta V_{dip,p'}$ from (S5.4) can be algebraically simplified to:

$\Delta V_{dip,p'}=\frac{\varepsilon_{w}-\varepsilon_{p}}{\varepsilon_{w}+\varepsilon_{p}}\Delta V_{dip,p},$ (S5.5)

for any choice of a dipole location () and the electrodes locations $\theta_{1,2}$. From (S5.5), the net voltage difference is thus:

$\Delta V_{dip}=\Delta V_{dip,p}+\Delta V_{dip,p'}=\frac{2\varepsilon_{w}}{\varepsilon_{w}+\varepsilon_{p}}\Delta V_{dip,p}.$ (S5.6)

In summary, the circular boundary simply rescales the differential dipole potential by a constant factor. The voltage rescaling does not influence our dipole localization algorithm, since it uses the relative signal intensities between multiple channels.

-end-


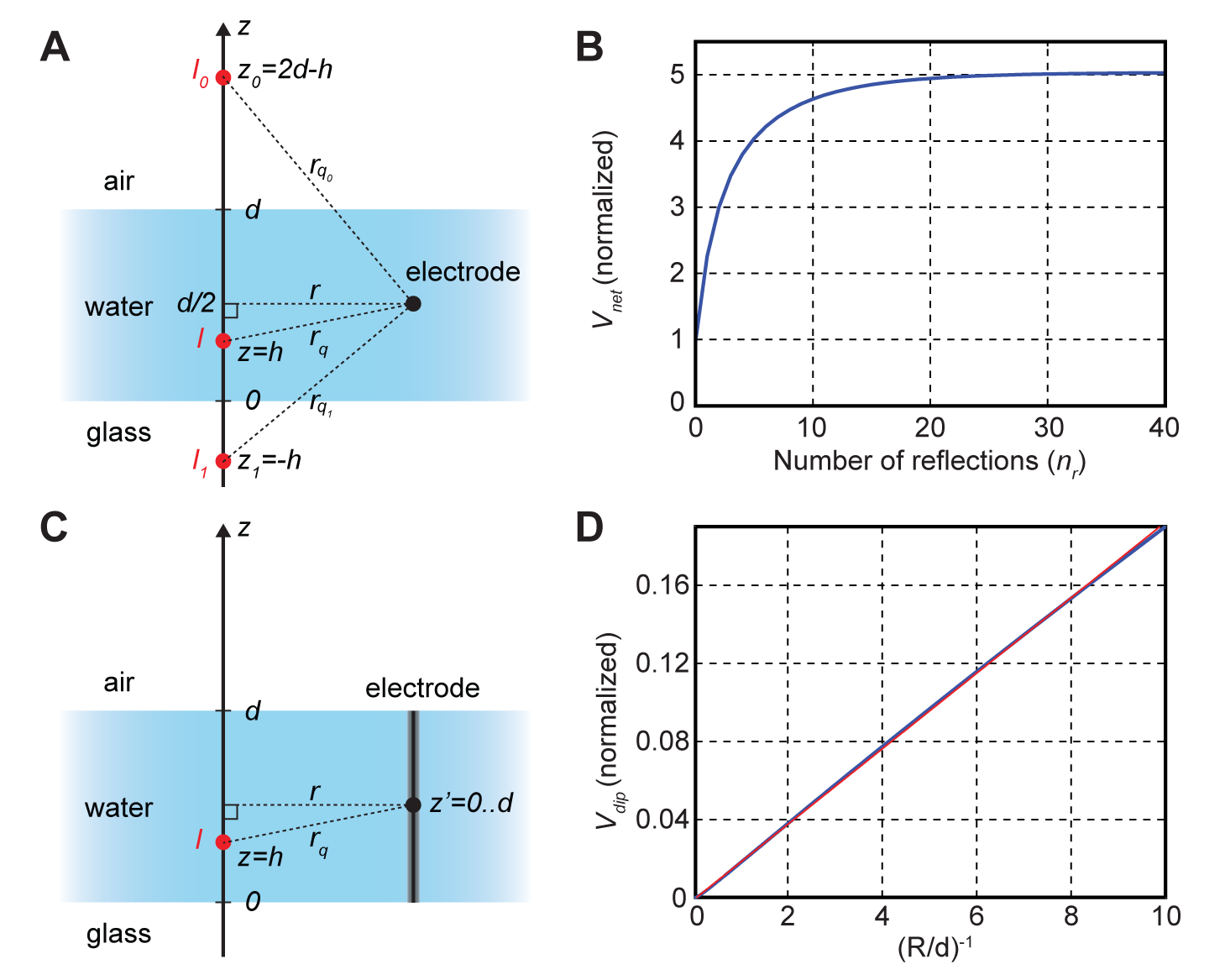


**Figure S1**. The method of image charges applied to the shallow body of water. (**A**) The image currents of the current source *I* created by the top and the bottom dielectric interfaces are shown up to the two first order reflections *I_0_* and *I_1_*. (**B**) The net potential (*V_net_*) due to the current source and its image currents is plotted as a function of the number of reflections (*n_r_*). *V_net_* was measured at the electrode at a distance *r=d*, and normalized to $I/4\pi\sigma_{w}d$ (*d*: depth of water). The current source was located at the height *d/2*. (**C**) The potential measured at the vertically oriented extended electrode was determined by averaging the potentials measured at different heights. (**D**) The numerically calculated potential of the vertically oriented electrode (*V_dip_*) is plotted in blue as a function of the normalized inverse distance (*R/d*)^-1^. The 2D ideal dipole voltage approximation is shown in red.

**
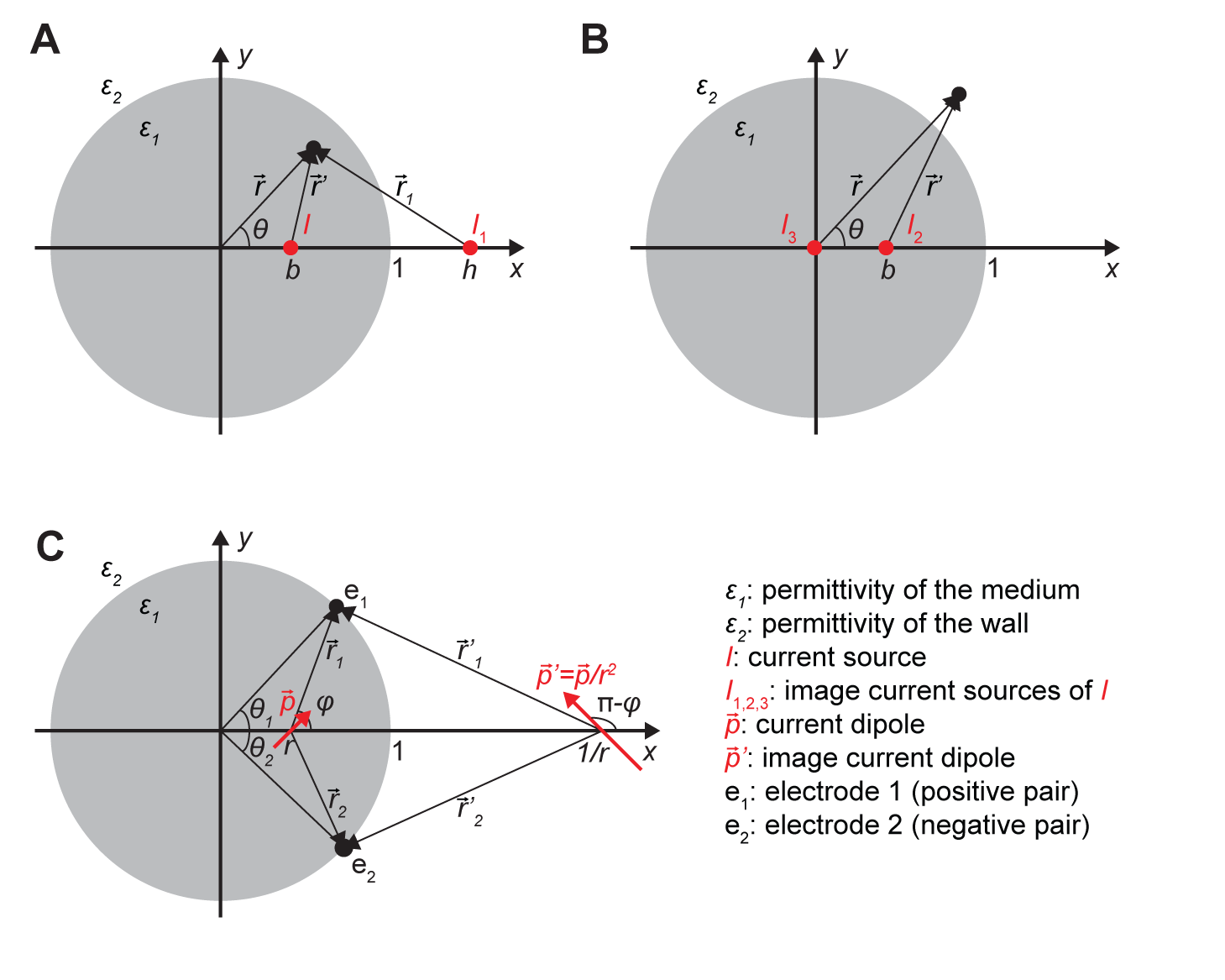
**

**Figure S2**. The method of image charges applied to the side circular boundary. (**A**) The current source (*I*) and its image source (*I_1_*) are shown for the field location inside of the circular region. (**B**) Two image sources (*I_2_, I_3_*) are shown for the field location outside of the circular region. (**C**) The image current dipole ($\vec{p}'$) location is shown to calculate the differential potential between the electrode pair (*e_1_*, *e_2_*).
