# Supplementary material for: Real-Time Localization of Moving Dipole Sources for Tracking Multiple Free-Swimming Weakly Electric Fish
Source: PLoS One. 2013 Jun 21;8(6):e66596. doi: 10.1371/journal.pone.0066596 (PMC3689756; doi:10.1371/journal.pone.0066596)
Supplement: Data S1 — includes a software demonstration package for the dipole tracking with a sample dataset and an instruction manual. (ZIP) [file pone.0066596.s007.zip › Software demo instructions.doc]

**Supplementary Text S2**

***Instructions for the dipole tracking demo***

# List of Files

| **File name** | **File format** | **Contents** |
| --- | --- | --- |
| demo_single.zip | Spike2 recording | Raw voltage recording from single fish (unzip will create *demo_single.smr* file) |
| demo_dyad.zip | Spike2 recording | Raw voltage recording from fish dyad (unzip will create *demo_dyad.smr* file) |
| measureRSI.s2s | Spike2 script | Measures received signal intensity (RSI) by measuring the EOD slopes and exports to Matlab |
| demo_single.mat | Matlab data | Spike2 data exported to Matlab |
| demo_dyad.mat | Matlab data | Spike2 data exported to Matlab |
| demo_single.mp4 | Video recording | Single fish infrared video recording |
| demo_dyad.mp4 | Video recording | Fish dyad infrared video recording |
| MASK.mat | Matlab data | Image mask to hide outside of the circular tank |
| demo.m | Matlab script | Dipole tracking demo code |
| output_single.mat | Matlab data | Dipole tracking result produced by the single fish tracking demo |
| output_dyad.mat | Matlab data | Dipole tracking result produced by the fish dyad tracking demo |

# Requirements

1. (Optional) CED Spike 2 (demo version available from http://ced.co.uk).
2. Mathworks Matlab.
3. Image processing toolbox for Matlab (to import and play the video recording).
4. Signal processing toolbox for Matlab.

# Instructions

1. Run demo.m and select which demo mode to run (single or dyad).
2. Watch the figure window displaying the tracking results.
3. Watch the animation illustrating the dipole tracking results superimposed with an infrared video recording.
4. Open the tracking output file (*output_single.mat* or *output_dyad.mat*).
